# Sequential domain assembly of ribosomal protein S3 drives 40S subunit maturation

**DOI:** 10.1038/ncomms10336

**Published:** 2016-02-02

**Authors:** Valentin Mitterer, Guillaume Murat, Stéphane Réty, Magali Blaud, Lila Delbos, Tamsyn Stanborough, Helmut Bergler, Nicolas Leulliot, Dieter Kressler, Brigitte Pertschy

**Affiliations:** 1 Institut für Molekulare Biowissenschaften, Universität Graz, Humboldtstrasse 50, 8010 Graz, Austria; 2 Unit of Biochemistry, Department of Biology, University of Fribourg, Chemin du Musée 10, CH-1700 Fribourg, Switzerland; 3 Laboratoire de Cristallographie et RMN Biologiques, UMR CNRS 8015, Université Paris Descartes, Sorbonne Paris Cité, Faculté de Pharmacie, 75006 Paris, France

## Abstract

Eukaryotic ribosomes assemble by association of ribosomal RNA with ribosomal proteins into nuclear precursor particles, which undergo a complex maturation pathway coordinated by non-ribosomal assembly factors. Here, we provide functional insights into how successive structural re-arrangements in ribosomal protein S3 promote maturation of the 40S ribosomal subunit. We show that S3 dimerizes and is imported into the nucleus with its N-domain in a rotated conformation and associated with the chaperone Yar1. Initial assembly of S3 with 40S precursors occurs via its C-domain, while the N-domain protrudes from the 40S surface. Yar1 is replaced by the assembly factor Ltv1, thereby fixing the S3 N-domain in the rotated orientation and preventing its 40S association. Finally, Ltv1 release, triggered by phosphorylation, and flipping of the S3 N-domain into its final position results in the stable integration of S3. Such a stepwise assembly may represent a new paradigm for the incorporation of ribosomal proteins.

## Abstract (web summary)

Ribosome biogenesis involves the hierarchical assembly of several proteins and RNA components. Here the authors describe a mechanism for ribosomal protein S3 incorporation into 40S ribosomal subunits that involves S3 dimerization and stepwise incorporation of two distinct S3 interaction domains coupled to release of ribosomal maturation factors.

## Graphical abstract

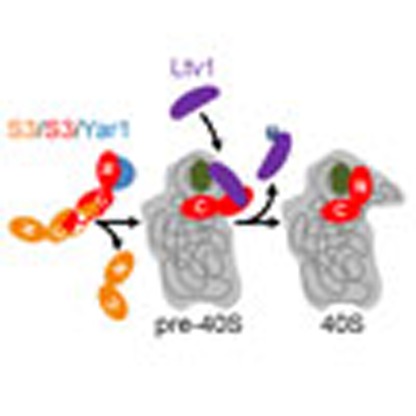

The assembly of ribosomal RNA (rRNA) and ribosomal proteins (r-proteins) into eukaryotic ribosomes is a highly complex, multi-step process, which requires the coordinated action of over 200 assembly factors and takes successively place in the nucleolus, nucleoplasm and cytoplasm. Most of the ∼80 r-proteins are assembled co-transcriptionally with a precursor rRNA, forming the large 90S particle (also termed SSU processome) that is subsequently separated into precursors of the large 60S (LSU) and the small 40S ribosomal subunit (SSU; reviewed in refs 1, 2, 3, 4). Although most non-ribosomal 40S assembly factors present in the SSU processome leave the biogenesis pathway again in the nucleus, a few factors (namely Ltv1, Enp1, Pno1, Nob1, Dim1, Tsr1, Rio2, Hrr25, Fap7 and Rrp12) accompany the pre-ribosomal 40S particle to the cytoplasm. After fulfilling their distinct tasks, they are progressively released and recycled back to the nucleus in a hierarchical order that is not entirely resolved so far^5^,^6^,^7^,^8^,^9^,^10^,^11^,^12^,^13^,^14^,^15^. Cytoplasmic 40S maturation events include the final rRNA processing step of 20S pre-rRNA into mature 18S rRNA by the endonuclease Nob1 (refs 16, 17, 18), which takes place during a translation-like cycle in 80S-like ribosomes after the transient joining of mature 60S subunits^10^,^19^,^20^.

A crucial structural reorganization within the 40S head domain, occurring before 20S pre-rRNA cleavage, involves the r-protein S3 (Rps3) and leads to the formation of the characteristic beak structure, shaped by protrusion of 18S rRNA helix 33 (ref. 8). Rps3 forms a ternary complex with the assembly factors Ltv1 and Enp1, which is salt-extractable from pre-ribosomal particles^8^. Cryo-electron microscopy and crosslinking data revealed the position of Ltv1 and Enp1 on the pre-40S solvent side^9^,^21^. Within this complex, Ltv1 presumably adopts an elongated shape bridging the gap between the 40S head (18S rRNA helix 41) and shoulder (18S rRNA helix 16) regions, and thereby preventing the opening of the mRNA entry channel. Beak formation coincides with the phosphorylation-dependent release of Ltv1 by the kinase Hrr25, which is supposed to be the initial step in cytoplasmic 40S maturation^7^,^8^,^15^. During these remodelling steps, the initially weakly associated, salt-extractable Rps3 adopts its final position and becomes stably bound to the 40S particle^8^; however, the mechanistic basis for this stable incorporation of Rps3 has remained elusive.

We have previously shown that, before its assembly into pre-ribosomal particles, Rps3 is associated with a specific chaperone, the ankyrin-repeat protein Yar1 (ref. 22). Recently, the co-crystal structure containing Rps3 and Yar1 revealed the organization of this complex^23^. Rps3 is composed of two globular domains (hereafter referred to as Rps3 N- and C-domain) followed by an unstructured C-terminal tail. The K homology N-domain of Rps3 binds to the central, structured core of Yar1 containing four ankyrin repeats. Compared to ribosome-bound Rps3, the structure of Rps3 in the Rps3/Yar1 complex shows two radical differences, which were both interpreted as non-physiological conformations formed as artefacts of crystallization^23^: (1) the Rps3 N-domain is rotated by ∼180° relative to the C-domain, and (2) the Rps3 C-domain dimerizes with a second Rps3 C-domain by domain swapping, thereby forming a tetrameric Rps3/Yar1 complex.

In this study, we report the solution structure of the Rps3/Yar1 complex by small-angle X-ray scattering (SAXS), revealing that dimerization of the Rps3 C-domain and the relative orientation of the N- versus the C-domain of Rps3 are not crystallization artefacts, but also occur in solution. Further, we show that Yar1-bound, non-ribosome-associated Rps3 also dimerizes *in vivo*. We demonstrate that Ltv1 replaces Yar1 as binding partner of the Rps3 N-domain, thereby preventing premature 40S incorporation of this domain. Initial assembly of Rps3 into pre-ribosomal particles is presumably mediated by its C-domain, thus, resolving the dimeric structure. A two-step mechanism, involving phosphorylation of Ltv1 and formation of critical contacts among Rps3, rRNA and the r-protein Rps20, eventually leads to the cytoplasmic release of Ltv1 and hence the stable integration of the Rps3 N-domain into 40S subunits. Taken together, we have unveiled the intricate mechanisms regulating the stable assembly of Rps3, which promotes the final steps of cytoplasmic 40S maturation.

## Results

### Rps3/Yar1 solution structure reveals Rps3 dimerization

Before assembly with ribosomal subunit precursors in the nucleus, Rps3 is bound by its specific chaperone Yar1, but how this interaction affects Rps3 incorporation into 40S precursors is currently unknown. To understand the molecular details of the transition from Yar1-bound Rps3 to ribosome-bound Rps3, we undertook a structural investigation of the Rps3/Yar1 complex in solution.

**Figure 1:**
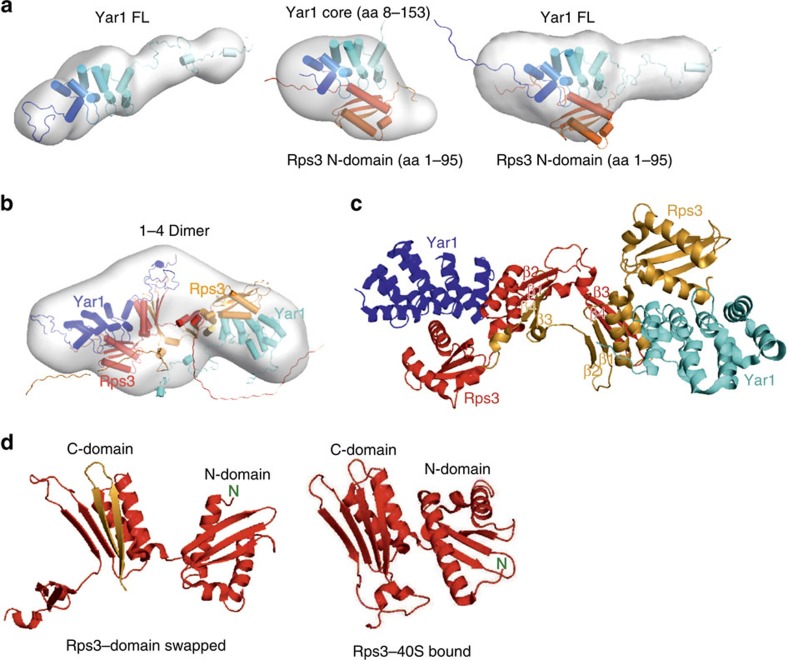
The Rps3 C-domain is rotated relative to the N-domain and dimerizes by domain swapping. (**a**) Model fit into a representative SAXS envelope for full-length Yar1 (FL), Rps3 N-domain (1–95)/Yar1 core (8–153) complex, Rps3 N-domain/Yar1-FL complex. Yar1 is coloured from blue (N terminus) to light blue (C terminus), Rps3 is coloured in orange. (**b**) Tetrameric arrangement of full-length Rps3/Yar1 complex. A representative envelope and the best fitting model of the dimeric conformations shown in Supplementary Fig. 2c extracted from the crystal structure (1–4 dimer) are shown. (**c**) Crystal structure of the 1–4 dimer (Protein Data Bank (PDB) 4BSZ). The same colours are used as in (**b**). The four β-sheets of the Rps3 C-domain were numbered to highlight that β-sheets 3 and 4 are swapped between the two Rps3 molecules. (**d**) Rps3 structure extracted from (**c**) with completion of the fold by the two swapped β-sheets (left panel). Domain swapping restores a similar fold of the C-domain as found in ribosome-bound Rps3 (PDB 3U5C, right panel). Comparing the two Rps3 conformations, the N-domain is rotated by ∼180° relative to the C-domain.

The crystal structure of the Rps3/Yar1 complex showed that the concave ankyrin-repeat surface of Yar1 binds the N-terminal α-helix of Rps3 (residues 14–29), while the Rps3 C-domain is in contact with the second α-helix of the fourth ankyrin-repeat^23^. *In vitro* binding assays confirmed that the very N-terminal region of Rps3 mediates the interaction with the ankyrin-repeat core domain of Yar1 (Supplementary Fig. 1). To address how the complex is organized in solution, thereby avoiding crystallization artefacts, and to model the N- and C-terminal extensions of Yar1 and Rps3 missing in the crystal structure (corresponding to ∼30% of the residues), we determined the solution structure of the complex by SAXS. The data obtained for the Rps3 N-domain (1–95)/Yar1 core (8–153) complex showed good agreement with a model constructed from the crystal structure (Fig. 1a; Supplementary Fig. 2a, middle panels; Supplementary Table 1). SAXS model structures of full-length Yar1 (Yar1-FL) confirmed the presence of unstructured extensions at the N and C termini; moreover, using this extended Yar1 structure, we could construct the Rps3 N-domain/Yar1-FL complex (Fig. 1a; Supplementary Fig. 2a and Supplementary Table 1). Next, we recorded SAXS data of the complex between the full-length Yar1 and Rps3 proteins. Surprisingly, we noticed that the molecular weight of the complex, as determined from the SAXS data, was twice as high as the sum of the calculated molecular weights of the individual components (Supplementary Table 1), indicating that the Rps3/Yar1 complex dimerizes. The fact that dimerization of the Rps3/Yar1 complex was only observed when full-length Rps3 was present, but not when the Rps3 C-domain was absent, suggested that the dimerization is mediated by the Rps3 C-domain. In the crystal structure, the Rps3/Yar1 complex is found in tetrameric arrangements (Supplementary Fig. 2b)^23^. To determine the conformation present in solution, we built three initial models representing the three dimeric forms of the Rps3/Yar1 complex that can be extracted from the crystal structure, corresponding to the dimers between complex 1 in Supplementary Fig. 2b and complexes 2, 3 and 4, respectively (denoted as 1–2 dimer, 1–3 dimer and 1–4 dimer hereafter; Supplementary Fig. 2b,c). The 1–4 dimer exhibited the conformation that fitted best to the SAXS data (Fig. 1b,c; Supplementary Fig. 2b,c). Interestingly, dimerization in this structure involves domain swapping of the two C-terminal β-strands of one Rps3 molecule with the equivalent β-strands of the opposing Rps3 molecule (Fig. 1c)^23^. Swapping of these two β-sheets restores a similar fold of the Rps3 C-domain as present in the ribosome-bound structure (Fig. 1d)^24^. However, the relative orientation between the Rps3 C- and N-domains in the Rps3/Yar1 complex markedly differs from the Rps3 conformation in the mature ribosome, with the N-domain rotated by ∼180° (Fig. 1d). Taken together, our experimental SAXS data reveal that Rps3 dimerization by domain swapping occurs in solution and moreover indicate that the relative orientation between the Rps3 N- and C-domains, which differs from the conformation in the ribosome by a ∼180° rotation (Fig. 1d)^24^, is not a crystallization artefact and might be physiologically relevant.

### Rps3 dimerizes *in vivo*

We previously conducted a random PCR mutagenesis of *RPS3* and screened for mutants showing a synthetic growth defect in combination with the *yar1*Δ mutant^22^. Five mutants were isolated, each resulting in two to three amino acid exchanges in Rps3 (Supplementary Fig. 3a,b). Notably, four of these mutants contained one exchange in residues within the C-domain forming contacts involved in domain swapping. On the basis of these genetic data, we considered that domain swapping and consequently dimerization of Rps3 might also occur *in vivo*.

**Figure 2:**
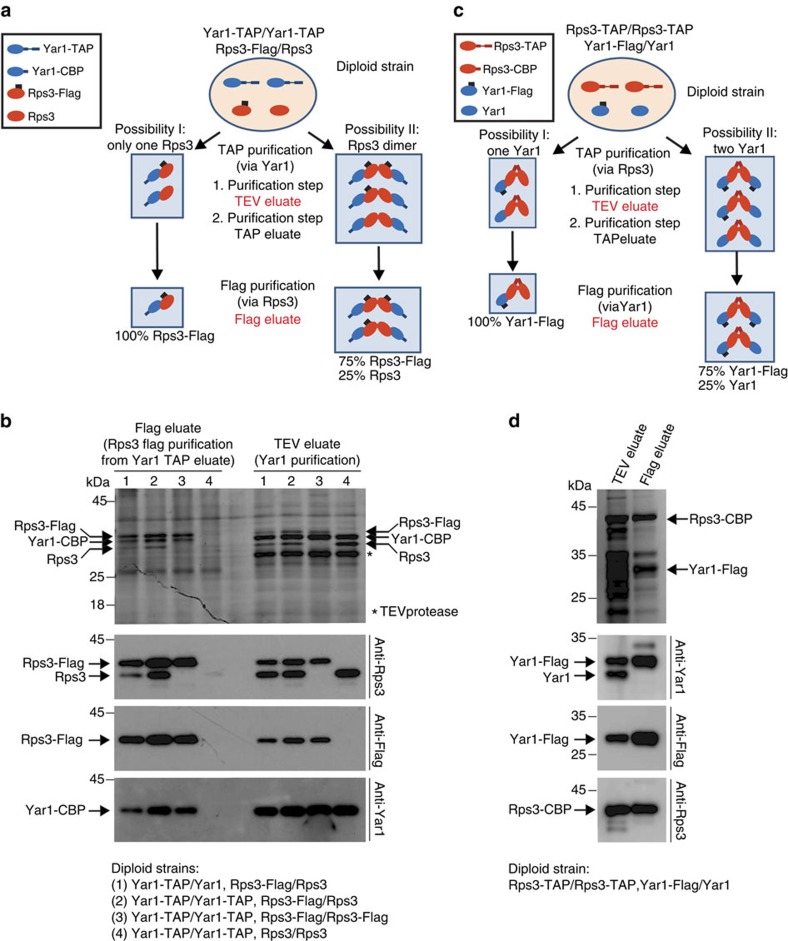
Rps3 dimerizes *in vivo*. (**a**) Experimental scheme of the purification shown in (**b**). The TAP-tag is composed of a protein A part, a TEV cleavage site and a calmodulin-binding protein (CBP) moiety. First, Yar1 is purified via protein A and eluted by the TEV protease, releasing Yar1 fused to CBP (TEV eluate). Then, Yar1 is purified via the CBP tag and eluted (TAP eluate). Finally, Rps3-Flag contained in the eluate is purified. While only Yar1-CBP and Rps3-Flag should be recovered in the case of a monomeric complex, the occurrence of dimeric complexes would also lead to co-purification of untagged Rps3. Fractions analysed in the experiment in (**b**) are indicated in red letters. (**b**) The indicated diploid strains (numbers 1–4) were subjected to the purification protocol outlined in (**a**). TEV eluates and Flag eluates were analysed by SDS–PAGE and Coomassie staining (upper part) as well as western blotting using the indicated antibodies (lower three panels). Note that using strains 1 and 2, both Rps3-Flag and untagged Rps3 are found in the Flag eluates, indicating dimerization of Rps3. In contrast, only one Yar1 was present in the complex, as revealed by the absence of untagged Yar1 in the TEV and Flag eluate of strain 1. When both *RPS3* alleles were Flag-tagged (strain 3), only Flag-tagged Rps3 was found in the eluate, showing that the recovery of untagged Rps3 with strains 1 and 2 is not a consequence of proteolytic removal of the Flag-tag. Furthermore, no Rps3 was recovered when both *RPS3* alleles were untagged (strain 4). (**c**) Experimental scheme of the Rps3-TAP followed by Yar1-Flag purification shown in (**d**). (**d**) The diploid *RPS3*-TAP/*RPS3*-TAP *YAR1*-Flag/*YAR1* strain was subjected to the purification protocol outlined in (**c**). TEV eluates and Flag eluates were analysed by SDS–PAGE and Coomassie staining (upper part) as well as western blotting (lower three panels). Note that in the first purification step (TEV eluate) both Yar1-Flag and Yar1 are co-purified, showing that both can form complexes together with Rps3. However, after subsequent purification of Yar1-Flag, no untagged Yar1 is recovered, corroborating that each complex contains only one Yar1 copy.

Next, we aimed to test whether Rps3 forms dimers in the Rps3/Yar1 complex *in vivo*, applying a strategy adapted to the low amounts of endogenous complex that can be purified from yeast cells. To this end, we used a diploid yeast strain with tandem affinity purification tag (TAP-tag) fusions of both *YAR1* alleles, and a Flag-tag fusion of one *RPS3* allele (while the second *RPS3* allele remained untagged; experimental scheme in Fig. 2a). We performed TAP of Yar1, followed by Flag purification of Rps3. If Rps3 occurs as a monomer in complex with Yar1, only Yar1-calmodulin-binding protein (Yar1-CBP) and Rps3-Flag should be recovered after this purification procedure (Fig. 2a, left panel). If, however, Rps3 dimerizes, Yar1-CBP and both Rps3-Flag and untagged Rps3 should be recovered (Fig. 2a, right panel). Indeed, we detected not only Rps3-Flag but also untagged Rps3 in the purification, confirming dimerization of Rps3 within the Rps3/Yar1 complex (Fig. 2b, strain 2). In addition, we tested whether two Yar1 molecules are present in the Rps3/Yar1 complex, as suggested by the SAXS analyses. To this end, we used a diploid strain with one *YAR1* allele TAP-tagged and the second allele untagged, together with one Flag-tagged and one untagged *RPS3* allele. In this setup, again both Rps3-Flag and untagged Rps3 were purified, while surprisingly only Yar1-CBP and no untagged Yar1 was found in the eluate, suggesting that *in vivo*, only one Yar1 is bound to an Rps3 dimer (Fig. 2b, strain 1). To further confirm this observation, we performed the inverse experiment, using a strain with both *RPS3* alleles TAP-tagged and one Flag-tagged, and one untagged *YAR1* allele (Fig. 2c,d). In this setup, Rps3-containing complexes (that is, ribosomes, pre-ribosomal particles and Rps3/Yar1 complex) are purified in the first step. As expected, both Yar1-Flag and Yar1 were recovered after this purification step (Fig. 2d). However, after subsequent Flag purification, only Yar1-Flag, but no untagged Yar1 was eluted, suggesting only one Yar1 molecule is present in each complex. We conclude that, *in vivo*, one Yar1 is bound to an Rps3 dimer (see also Discussion).

### Ltv1 replaces Yar1 as a binding partner of the Rps3 N-domain

Next, we aimed to further follow the ribosome assembly path of Rps3. Inspection of crosslinking data, and the pre-40S cryo-electron microscopy structure revealed that assembly of the Rps3 N-domain in its mature conformation would clash with binding of the 40S assembly factor Ltv1 at helix 41 (around 18S rRNA nucleotide C1490)^9^,^21^,^24^, suggesting that the Rps3 N-domain should be displaced within pre-40S particles. Moreover, footprinting analyses revealed that in pre-40S particles, the nucleotides in helix 41 contacted by the N terminus of Rps3 are more reactive to chemical modification, further supporting that Rps3 is not bound at that site in pre-40S particles^25^.

These data, together with the fact that Yar1 binds to the Rps3 N-domain, suggest that initial 40S incorporation of Rps3 most likely occurs via its C-domain, which represents the main rRNA-binding surface of Rps3 (refs 23, 24). Considering that mature 40S subunits contain only one copy of Rps3, the second Rps3 copy in the Rps3/Rps3/Yar1 complex is presumably released upon ribosome assembly. Consistently, the amounts of Rps3 relative to other r-proteins were equal in purified pre-ribosomal particles and mature 40S subunits, suggesting that the stoichiometry of Rps3 remains the same during the course of pre-40S maturation (Supplementary Fig. 4a).

Since we were unable to detect significant amounts of Yar1 in purified pre-ribosomal particles and as Yar1 sediments in the soluble fraction of sucrose gradients^22^,^26^, Yar1 likely dissociates from Rps3 before, upon, or shortly after pre-ribosome assembly of Rps3. Hence, on pre-ribosomal particles, other assembly factors might bind the Rps3 N-domain subsequently to Yar1. Rps3 was shown to form a salt-extractable complex with Ltv1 and Enp1 in pre-40S particles^8^. Thus, we considered Ltv1 and Enp1 as candidates for successors of Yar1 in Rps3 N-domain binding.

**Figure 3:**
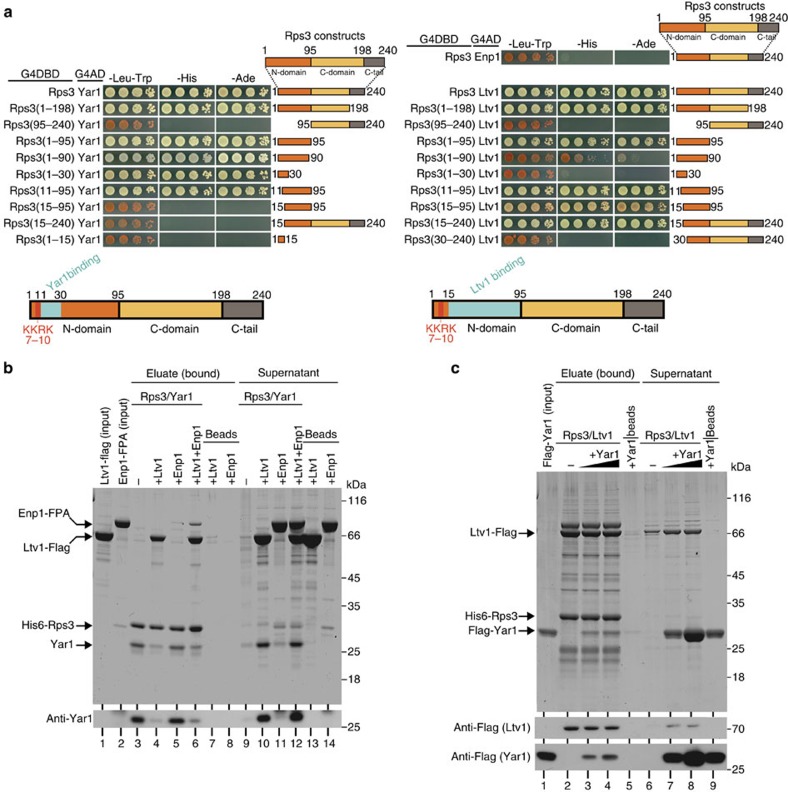
Ltv1 releases Yar1 upon binding to the Rps3 N-domain. (**a**) Yar1 and Ltv1 have overlapping binding sites on the Rps3 N-domain. Different Rps3 truncations fused to the Gal4 DNA-binding domain (G4DBD) were tested for Y2H interaction with Yar1 (left panel), Enp1 or Ltv1 (right panel) fused to the Gal4 activation domain (G4AD). Growth on -His and -Ade plates, as well as white colony colour on -Leu-Trp plates, is indicative of a strong interaction. Both Yar1 and Ltv1 interact with N-terminal Rps3 fragments, whereas C-terminal Rps3 fragments do not mediate Y2H interaction with Yar1 or Ltv1. Below, the deduced binding domains, highlighted in blue colour, for each protein are illustrated schematically. (**b**) Ltv1 competes with Yar1 for Rps3 binding. Purified His6-Rps3/Yar1 complex immobilized on Ni-NTA agarose (and as control empty Ni-NTA agarose beads) was incubated either with buffer or with an ∼6-fold molar excess (over Rps3) of purified Ltv1-Flag and/or Enp1-FPA (Flag-ProteinA). Supernatants of the incubation and eluates (containing His6-Rps3 and bound proteins) were analysed by SDS–PAGE and Coomassie staining or western blotting. (**c**) Yar1 competes only inefficiently with Ltv1 for Rps3 binding. Purified His6-Rps3/Ltv1-Flag complex immobilized on Ni-NTA agarose (and empty beads as negative control) was incubated either with buffer or with an ∼1.5-fold (lanes 3 and 7) or 6-fold (lanes 4 and 8) molar excess of purified Flag-Yar1. Eluates containing His6-Rps3 and bound proteins and supernatants were analysed by SDS–PAGE and Coomassie staining or western blotting.

Yeast two-hybrid (Y2H) analyses revealed a strong interaction between Rps3 and Ltv1 (Fig. 3a, right panel)^27^, while Enp1 did not interact with Rps3 (Fig. 3a, right panel). Moreover, Rps3 and Ltv1 showed a robust *in vitro* interaction (Supplementary Fig. 4b)^7^. Interaction domain mapping by Y2H identified a large fragment (amino acids 15–95) of the Rps3 N-domain to be both necessary and sufficient for Ltv1 binding (Fig. 3a, right panel). Hence, Ltv1 and Yar1 both interact with the Rps3 N-domain and have partially overlapping binding sites. To further assess whether Yar1 and Ltv1 compete for the same binding site on Rps3, we performed *in vitro* competition assays (Fig. 3b,c). Indeed, upon incubation of the Rps3/Yar1 complex, immobilized on Ni-NTA agarose via His6-Rps3, with purified Ltv1-Flag, Ltv1 bound to Rps3 and was present in the eluate, while Yar1 was released into the supernatant (Fig. 3b, lanes 4 and 10). In contrast, Enp1 was not able to bind Rps3 and compete Yar1 off (Fig. 3b, lanes 5 and 11). However, when Ltv1 was also present, a ternary Rps3/Ltv1/Enp1 complex was established, leading to Yar1 release (Fig. 3b, lanes 6 and 12). Next, we immobilized Rps3/Ltv1 complex on Ni-NTA agarose via His6-Rps3 and incubated the complex with purified Flag-Yar1. Although some Yar1 binding to Rps3 (Fig. 3c, lanes 3 and 4) and some Ltv1 release (Fig. 3c, lanes 7 and 8) was observed, Yar1 was clearly less efficient in competing Ltv1 off Rps3 than Ltv1 was in competing Yar1 off (Fig. 3b,c). We conclude that Ltv1 has a higher affinity for Rps3 than Yar1. Interestingly, a recent study indicates that *in vitro* Rps3 is efficiently loaded onto pre-ribosomal particles only in the presence of Ltv1 (ref. 7). We suggest that the Rps3 N-domain is handed over from the Rps3/Yar1 complex to Ltv1 shortly before, during or shortly after Rps3 assembly. Eventually, Ltv1 needs to be released again in order to allow for the final incorporation of the Rps3 N-domain into the 40S ribosomal subunit.

### Ltv1 is released upon phosphorylation at S336, 339 and 342

**Figure 4:**
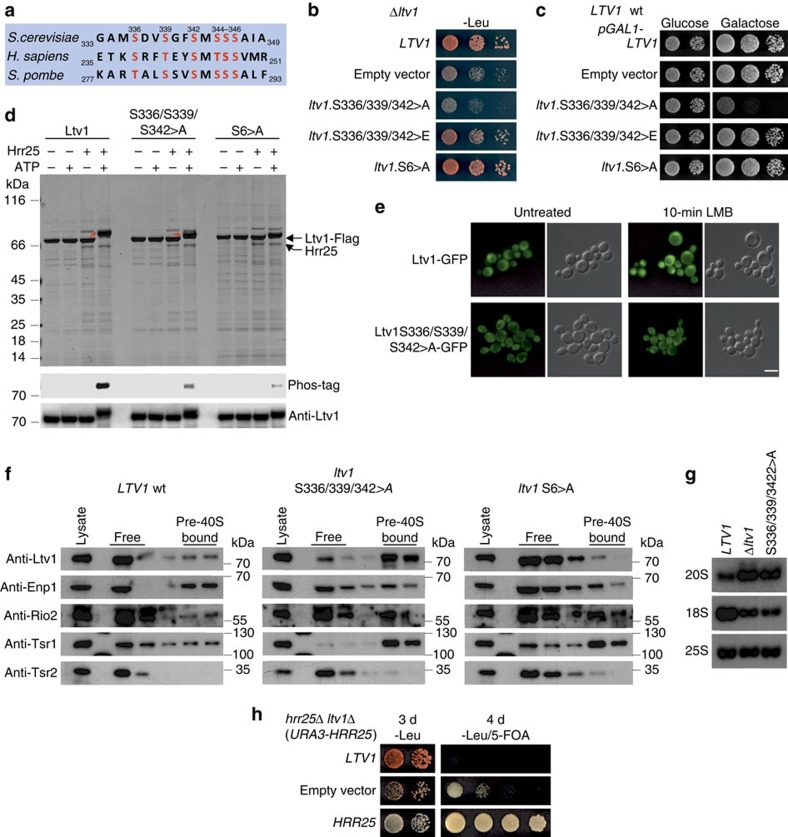
Cytoplasmic Ltv1 release is triggered by phosphorylation of serines 336, 339 and 342, and is essential for cell growth. (**a**) Sequence alignment reveals a conserved serine/threonine-rich motif in Ltv1 as putative target for phosphorylation by Hrr25/CK1δ/ɛ. (**b**) Growth phenotypes of the indicated *ltv1* phosphomutant strains at 23 °C (3-day (d) incubation). (**c**) Wild-type *LTV1* and *ltv1* phosphomutant alleles were overexpressed from the galactose inducible *GAL1-10* promoter in an *LTV1* wild-type background. Growth was examined after incubation on glucose (repressed) or galactose (induced) containing plates at 23 °C for 3 and 7 d, respectively. (**d**) The indicated Flag-tagged Ltv1 proteins were incubated with Hrr25 in the presence or absence (±) of ATP. Samples were analysed by SDS–PAGE and Coomassie staining (upper panel) or western blotting (lower two panels). Phosphorylated Ltv1 was detected with the phosphate-binding reagent Phos-tag and is also recognizable as a slower migrating band. (**e**) The localization of wild-type Ltv1-GFP or Ltv1(S336A/S339A/S342A)-GFP was examined in LMB-sensitive *crm1* mutant cells before and after 10 min of incubation with LMB. Note that due to the *crm1* mutation, Ltv1-GFP, but not the phosphomutant, partially localizes in the nucleus already in untreated cells. After 10 min of treatment, a strong nuclear accumulation of Ltv1-GFP can be observed, while the phosphomutant protein is still localized in the cytoplasm. Scale bar, 5 μm. (**f**) Cell lysates of wild-type *LTV1* or *ltv1* phosphomutant strains were analysed by sucrose gradient centrifugation, and the upper fractions of the gradients containing soluble proteins (free) to 40S subunits (pre-40S bound) were analysed by western blotting. Tsr2 was detected as marker for the soluble fraction. (**g**) Ltv1 phosphorylation is required for the final rRNA processing step. Indicated strains were analysed by northern blotting with probes directed against immature 20S and mature 18S and 25S rRNA species. (**h**) Phosphorylation-dependent pre-40S release of Ltv1 is the essential cellular function of Hrr25. A *hrr25*Δ *ltv1*Δ strain with wild-type *HRR25* on a *URA3*-plasmid was transformed with vector or plasmids carrying *LTV1* or *HRR25*. The ability to lose the *URA3*-*HRR25* plasmid was assessed on synthetic dextrose (SD) -leu plates containing 5-Fluoroorotic acid (5-FOA) incubated at 30 °C for 4 d. Although a *hrr25*Δ strain is inviable when *LTV1* is present, the strain is viable in the absence of *LTV1*.

Cytoplasmic release of Ltv1 requires its phosphorylation by the kinase Hrr25 (CK1δ/ɛ in human cells), and is a prerequisite for final 40S maturation steps such as 20S to 18S rRNA processing, beak formation and the formation of 80S-like ribosomes^7^,^8^,^15^. To further characterize this event, we attempted to identify the target site(s) within Ltv1 that are phosphorylated by Hrr25. Alignment of the Ltv1 protein sequence from different species revealed a conserved serine/threonine-rich motif from amino acids 336 to 346, resembling the consensus sequence recognized by CK1 (Fig. 4a)^28^,^29^. We substituted several different combinations of serines within this motif to alanine, and assessed the growth phenotypes of the resulting mutants (Supplementary Fig. 5a). The strongest growth defect was observed after combined substitution of the three serines S336, S339 and S342 to alanine. In contrast, substitution of the same serine residues to glutamate, which mimics phosphorylation, supported almost wild-type growth (Fig. 4b; Supplementary Fig. 5a). Moreover, overexpression of Ltv1(S336A/S339A/S342A), but not of Ltv1(S336E/S339E/S342E), resulted in a dominant-negative growth phenotype (Fig. 4c; Supplementary Fig. 5b). *In vitro* phosphorylation of Ltv1(S336A/S339A/S342A) by Hrr25 was clearly decreased compared to wild-type Ltv1, verifying these residues as the major Ltv1-phosphorylation sites (Fig. 4d; Supplementary Fig. 5c). While this manuscript was in preparation, the same residues were also reported by another study to be the main Ltv1-phosphorylation sites^7^. Ltv1 mutant protein with serine to alanine substitutions of all six serines from S336 to S346 (hereafter termed Ltv1(S6A)) was even less phosphorylated revealing that, in addition to S336, S339 and S342, also S344, S345 and S346 can be used as phosphorylation targets (Fig. 4d). However, the *ltv1*(S6A) mutant showed normal growth and no dominant-negative effect upon overexpression (Fig. 4b,c), suggesting that the multiple mutations lead to a looser association with 40S particles and allow the spontaneous dissociation of the protein without phosphorylation.

**Figure 5:**
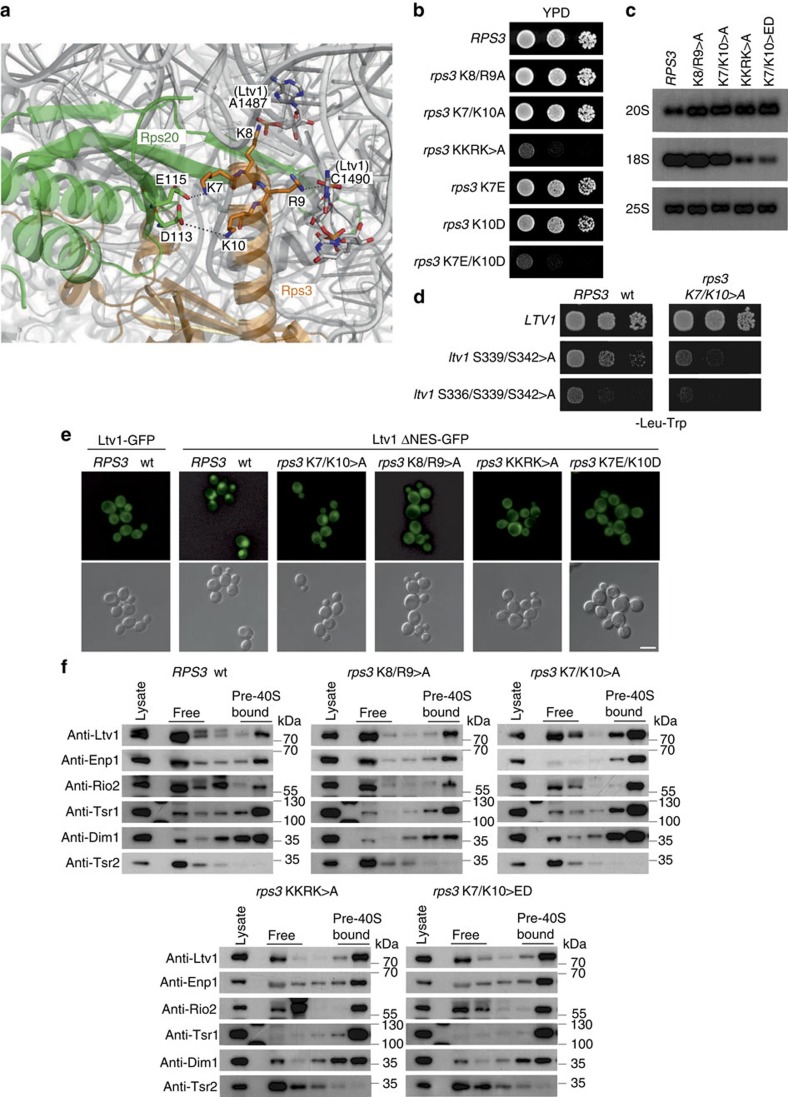
Assembly of the Rps3 N-domain releases Ltv1 from pre-40S particles and is required for final 40S maturation steps. (**a**) N-terminal Rps3 residues form ionic contacts with negatively charged Rps20 residues and with rRNA nucleotides A1487 and C1490 around the rRNA-binding site of Ltv1 in helix 41 (PDB 3U5B and 3U5C). (**b**) Residues of the Rps3 N-domain interacting with Rps20 and rRNA are required for optimal cell growth. Growth phenotypes of the indicated *rps3* alleles harbouring amino acid substitutions within the N-terminal residues K7, K8, R9 and K10 were examined after incubation at 23 °C for 3 days (d). (**c**) N-terminal *rps3* mutants display 20S pre-rRNA processing defects. The amounts of immature 20S and mature 18S rRNA in the indicated *rps3* mutants were analysed by northern blotting. (**d**) *rps3*(K7A/K10A) has a synthetically enhanced growth phenotype with *ltv1*-phosphorylation mutants. The genetic interaction between the indicated alleles was examined after incubation at 23 °C for 4 d. (**e**) The localization of Ltv1ΔNES-GFP was examined in cells expressing the indicated *rps3* alleles. Note that, in contrast to wild-type Ltv1-GFP, Ltv1ΔNES-GFP displays a strictly nuclear localization in *RPS3* cells that is shifted to the cytoplasm in the *rps3* mutants. Scale bar, 5 μm. (**f**) *rps3* mutants accumulate Ltv1 and further cytoplasmic assembly factors on pre-40S particles. Lysates of the indicated *rps3* mutants were analysed by 7–45% sucrose gradient centrifugation, and the upper fractions of the gradients containing soluble proteins (free) to 40S subunits (pre-40S bound) were analysed by western blotting with the indicated antibodies.

Next, we addressed whether cytoplasmic release of Ltv1(S336A/S339A/S342A) from pre-40S particles is impaired. Ltv1 has a cytoplasmic steady-state localization in wild-type cells (Fig. 5e), however, inhibition of nuclear export by leptomycin B (LMB) treatment of an LMB-sensitive *crm1* mutant resulted in a substantial nuclear accumulation of Ltv1-green fluorescent protein (Ltv1-GFP) already after 10 min (Fig. 4e). A similar accumulation of Ltv1-GFP was also observed when translation was blocked before LMB treatment by cycloheximide (CHX) addition, indicating that the nuclear accumulation is mainly due to recycling and not to neosynthesis of Ltv1-GFP (Supplementary Fig. 5e). In contrast, Ltv1(S336A/S339A/S342A)-GFP remained cytoplasmic after 10 min of LMB treatment. Some nuclear accumulation was only detected after 60 min, indicating a delay in recycling of Ltv1 back into the nucleus, hence suggesting a cytoplasmic pre-40S release defect (Fig. 4e; Supplementary Fig. 5e). Contrarily, the Ltv1(S6A) protein showed the same localization as wild-type Ltv1, indicating that cytoplasmic release was not impaired for this protein (Supplementary Fig. 5e).

The cytoplasmic release defect of the Ltv1(S336A/S339A/S342A) phosphomutant was further confirmed by sucrose gradient centrifugation, which revealed increased levels of the mutated Ltv1 sedimenting in the 40S fractions of the gradient, whereas wild-type Ltv1 and Ltv1(S6A) were predominantly found in the soluble fraction (Fig. 4f). Noticeably, the assembly factors Rio2 and Tsr1 also accumulated on pre-ribosomes in the *ltv1*(S336A/S339A/S342A) mutant (Fig. 4f), supporting the idea that the phosphorylation-dependent Ltv1 release is a prerequisite for downstream cytoplasmic maturation steps. In addition, likewise to an *ltv1*Δ strain, the mutant was inhibited in the final rRNA processing step, as revealed by the accumulation of immature 20S pre-rRNA and the reduction of mature 18S rRNA levels (Fig. 4g). Moreover, also other *ltv1* mutants harbouring serine to alanine exchanges, but not *ltv1*(S6A), displayed rRNA processing defects (Supplementary Fig. 5d).

The kinase Hrr25 is essential for yeast viability, and is involved in a wide range of cellular processes (see for example, refs 30, 31, 32). Since we observed severe defects for the *ltv1*-phosphorylation mutants, we reasoned that the role of Hrr25 in Ltv1 release might be particularly important. Indeed, *HRR25* became dispensable in an *ltv1* deletion strain (Fig. 4h). While this manuscript was in preparation, similar effects were reported upon shutdown of Hrr25 expression in *ltv1* deletion strains^7^. Hence, Ltv1 phosphorylation and its subsequent pre-40S release is the essential cellular function of Hrr25.

### 40S maturation requires formation of Rps3 N-domain contacts

As long as Ltv1 is bound to 18S rRNA helix 41, this part of the rRNA is not available for binding of the Rps3 N-domain via arginine 9 (R9) and lysine 8 (K8; Fig. 5a)^21^,^24^. To assess the physiological relevance of these Rps3-rRNA contacts, we analysed the phenotypes of mutants carrying exchanges within these residues. In addition, we mutated the adjacent lysine 7 (K7) and lysine 10 (K10), which engage in interaction with the r-protein Rps20 (Fig. 5a). Substitution of K8 and R9 (rRNA contacts) or K7 and K10 (Rps20 contacts) to alanine did not result in substantial growth defects, while combined substitution of all four residues to alanines (KKRK>A) resulted in significantly reduced cell growth (Fig. 5b). Remarkably, an even more severe growth defect was observed after mutating K7 and K10 to negatively charged glutamate and aspartate, respectively; thereby inducing a repulsion from the interacting negatively charged glutamate (E115) and aspartate (D113) residues of Rps20 (Fig. 5a,b). We have previously reported that the N-terminal KKRK-motif (amino acids 7–10) within Rps3 constitutes a functional nuclear localization signal (NLS)^22^. However, as fusion of the strong SV40-NLS to the *rps3* mutant alleles did not rescue the growth defects, the observed growth phenotypes can be attributed to the loss of interactions with 18S rRNA and Rps20, rather than to a nuclear import defect (Supplementary Fig. 6). Further analyses revealed that *rps3*(KKRK>A) and *rps3*(K7E/K10D) strains displayed severe rRNA processing defects, as evidenced by the accumulation of unprocessed 20S pre-rRNA and the decrease of mature 18S rRNA levels (Fig. 5c). Hence, the formation of the ionic contacts between Rps3 and Rps20, as well as between Rps3 and rRNA, is not only necessary for the integrity of mature 40S subunits, but appears to be also required for 40S subunit maturation.

### Rps3 N-domain assembly releases Ltv1 from pre-40S particles

Notably, we observed a synthetically enhanced growth defect of the *rps3*(K7A/K10A) mutant with *ltv1*-phosphorylation mutants, raising the idea that, in addition to Ltv1 phosphorylation, also the final assembly of the Rps3 N-domain might be involved in releasing Ltv1 from pre-ribosomal particles (Fig. 5d). To address the role of Rps3 residues K8, R9, K7 and K10 in Ltv1 release, we made use of a GFP fusion reporter construct containing a deletion of the recently reported C-terminal nuclear export sequence (NES) of Ltv1 (ref. 27). In contrast to the localization of wild-type Ltv1-GFP, Ltv1ΔNES-GFP strictly localized to the nucleus of *RPS3* wild-type cells (Fig. 5e). Strikingly, the localization shifted to the cytoplasm when monitored in the *rps3* assembly mutants, hence revealing a defect in pre-40S release of Ltv1 (Fig. 5e). While *rps3*(K7A/K10A) and *rps3*(K8A/R9A) mutants showed a partial mislocalization of the Ltv1ΔNES-GFP reporter, the localization was completely shifted to the cytoplasm in the *rps3*(KKRK>A) and *rps3*(K7E/K10D) mutants. Furthermore, these *rps3* mutants showed increased amounts of Ltv1 in the 40S fraction after sucrose gradient centrifugation (Fig. 5f). Remarkably, the assembly factors Enp1, Rio2 and Tsr1 also accumulated on pre-40S particles (Fig. 5f). We conclude that the residues of the Rps3 N-domain involved in interaction with 18S rRNA H41 and with Rps20 are required for efficient Ltv1 release from pre-40S particles, thereby permitting progression of cytoplasmic 40S maturation.

**Figure 6:**
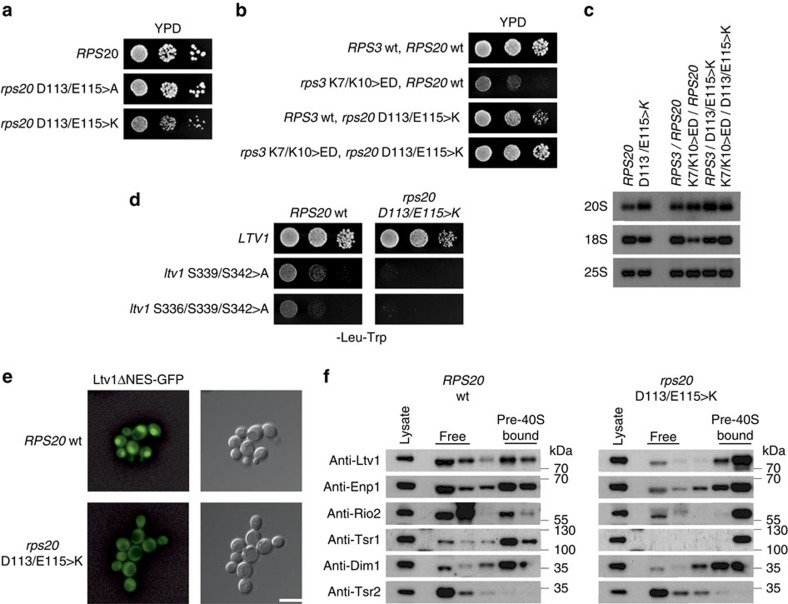
Cytoplasmic Ltv1 release is inhibited in the *rps20*(D113K/E115K) mutant. (**a**) Growth phenotypes of wild-type *RPS20* and *rps20*(D113K/E115K) mutant after incubation at 23 °C for 3 days (d). (**b**) Reversion of the charge repulsion of *rps3*/*rps20* mutants restores cell growth to wild-type rates. The growth phenotypes of the indicated mutants were assessed after incubation at 23 °C for 3 d. (**c**) The 20S pre-rRNA processing defect of *rps3*(K7E/K10D) and *rps20*(D113K/E115K) single mutants is partially rescued in the *rps3*/*rps20* double mutant. The indicated *rps3* and *rps20* mutants were analysed by northern blotting. (**d**) Synthetically enhanced growth phenotype of *rps20*(D113K/E115K) with *ltv1* phosphomutants. The genetic interaction between the indicated alleles was assessed after incubation at 23 °C for 4 d. (**e**) Ltv1ΔNES-GFP mislocalizes to the cytoplasm in *rps20*(D113K/E115K) cells. The localization of Ltv1ΔNES-GFP was examined in *RPS20* and *rps20*(D113K/E115K) cells. Scale bar, 5 μm. (**f**) Lysates of *RPS20* and *rps20*(D113K/E115K) cells were analysed by 7–45% sucrose gradient centrifugation, and the upper fractions of the gradients containing soluble proteins (free) to 40S subunits (pre-40S bound) were analysed by western blotting with the indicated antibodies.

If the assembly defects displayed by the *rps3*(K7E/K10D) mutant were due to the inability in forming the ionic contacts with Rps20, similar phenotypes should be observed when the contacts are disrupted by mutating *RPS20*. Indeed, an *rps20*(D113K/E115K) mutant displayed a slow-growth phenotype and an Ltv1 release defect (Fig. 6a,e,f). Furthermore, the *rps20* mutant was genetically linked to *ltv1*-phosphorylation mutants (Fig. 6d) and showed a 20S pre-rRNA processing defect (Fig. 6c). Strikingly, when the contact between Rps3 and Rps20 was restored by simultaneously mutating both interaction sites (*rps3*(K7E/K10D)/*rps20*(D113K/E115K)), thus reverting the charge repulsion, cells almost grew at wild-type rates again (Fig. 6b) and the rRNA processing defect was partially rescued (Fig. 6c). Notably, as this *rps3-rps20* double mutation restores the Rps3-Rps20 interaction while leaving the Rps3-NLS non-functional, the normal growth suggests that apart from the import route involving this N-terminal NLS, also additional, redundant Rps3 import pathways have to exist. We conclude that the phenotypes observed in *rps3* and *rps20* mutants can be attributed to the inability to form the contact between Rps3 and Rps20. This interaction is necessary for Ltv1 release, but is likely also important for the function of the mature 40S subunit.

### Ltv1 is positioned between the Rps3 N-domain and Rps20

**Figure 7:**
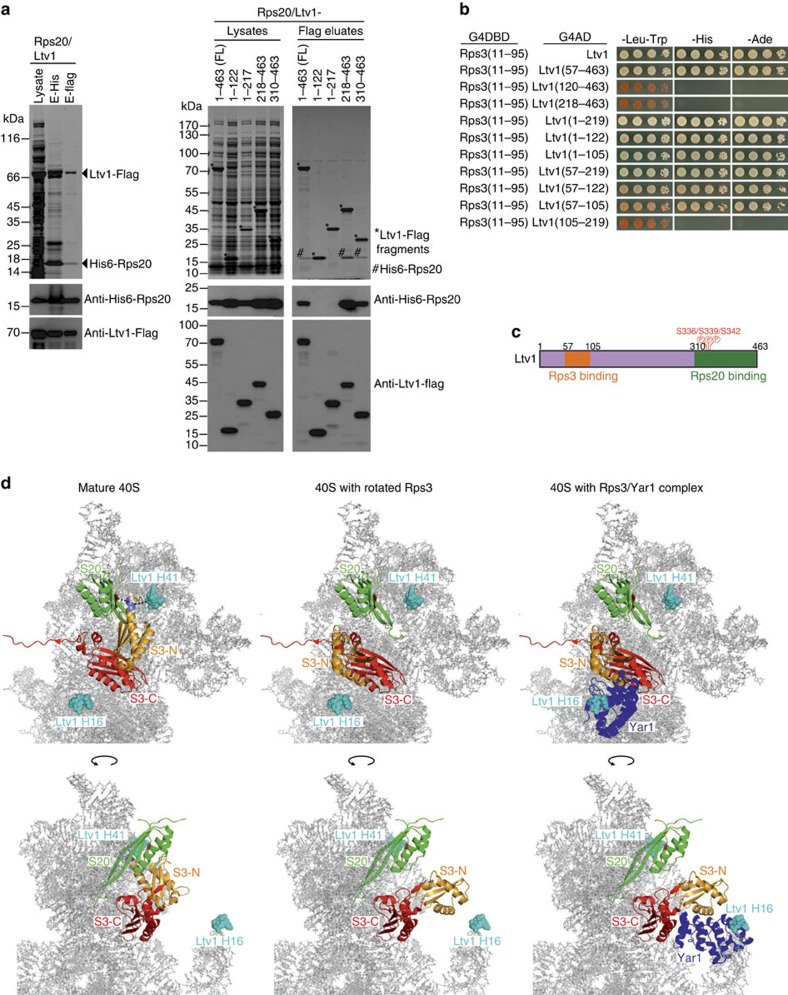
Ltv1 is clamped between Rps20 and the rotated Rps3 N-domain. (**a**) The C-terminal part of Ltv1 binds Rps20. Left panel: His6-Rps20 and Ltv1-Flag were co-expressed in *E. coli*. In a first purification step, His6-Rps20 was pulled down with Ni-NTA agarose and the obtained eluate was subjected to a second purification step with anti-Flag agarose. Cell lysate, His and Flag eluates were analysed by SDS–PAGE and Coomassie staining or western blotting. Right panel: after co-expression of His6-Rps20 with the indicated Flag-tagged N- and C- terminal Ltv1 truncations in *E. coli*, cell lysates were subjected to affinity purification with anti-Flag agarose. Cell lysates and Flag eluates were analysed by SDS–PAGE and Coomassie staining or western blotting. (**b**) N-terminal Ltv1 fragments interact with Rps3. Y2H domain mapping of Ltv1 truncations fused to the G4DBD and Rps3 fused to the G4AD reveal amino acids 57–105 as the Ltv1 interaction surface for Rps3 binding. (**c**) Rps3 and Rps20 interaction regions of Ltv1. Phosphorylated Ltv1 serines are indicated. (**d**) Initial incorporation of Rps3 in its rotated conformation allows pre-ribosomal assembly of Ltv1. Rps3 protein from the Rps3/Yar1 crystal structure (PDB 4BSZ, Fig. 1d, left panel) was modelled into the 40S crystal structure (PDB 3U5B and 3U5C) by superposition of the Rps3 C-domains. Compared to the position of the Rps3 N-domain within the mature ribosome (left panels), the Rps3 N-domain in the modelled conformation protrudes away from the 40S surface (middle panels) and is accessible for interaction with Yar1 (right panel) or other assembly factors. This N-domain rotation is compatible with Ltv1 binding at its rRNA-binding site at helix 41, which is occupied by the N-terminal Rps3 α-helix in the mature 40S subunit. In contrast, the positioning of Yar1 is incompatible with Ltv1 binding to helix 16, further suggesting Yar1 release upon Ltv1 binding. The Rps3 N- and C-domains are shown in orange and red, respectively, the Ltv1-rRNA crosslink sites at helix 16 and helix 41 are shown as spheres coloured in cyan, Rps20 is displayed in green and Yar1 in blue.

On the basis of our results linking the cytoplasmic Ltv1 release to the association of the Rps3 N-domain with Rps20, and considering that the Ltv1-rRNA crosslinking site in helix 41 is physically close to Rps20, we reasoned that in pre-40S particles Ltv1 might be in direct contact not only with Rps3 but also with Rps20. Indeed, following a two-step affinity purification, we detected a robust *in vitro* interaction between Ltv1 and Rps20 (Fig. 7a, left panel). We hypothesized that Ltv1 is clamped between Rps3 and Rps20 on pre-40S ribosomal subunits, thereby preventing an interaction between the two r-proteins at a too early maturation stage. To determine the orientation of Ltv1 on pre-40S particles, we mapped the Rps3 and Rps20 interaction sites of Ltv1. Consistent with a previous study^27^, we found a fragment close to the Ltv1 N terminus (amino acids 57–105) to mediate the Y2H interaction with the Rps3 N-domain (Fig. 7b,c). In contrast, the *in vitro* interaction with Rps20 is mediated by the C-terminal part of Ltv1, with amino acids 310–463 being sufficient for interaction with Rps20 (right panel in Fig. 7a and Fig. 7c). Hence, considering the proposed elongated structure of Ltv1, spanning from rRNA helix 16 to helix 41, there has to be a significant distance between the Rps3 N-domain and Rps20 in pre-40S particles.

To predict the position of the Rps3 N-domain in pre-40S particles, we considered the possibility that Rps3 or the Rps3/Yar1 complex is incorporated into early ribosomal precursors with the rotated domain arrangement that we observed in the Rps3/Yar1 solution structure. We modelled the Rps3/Yar1 complex both into the 40S crystal structure and into the pre-40S cryo-electron microscopy structure by assuming the same orientation of the Rps3 C-domain as in mature 40S subunits (Fig. 7d; Supplementary Fig. 7). Indeed, the Rps3 N-domain protruded away from the 40S surface in these models, preventing it from interaction with rRNA and Rps20. This conformation might allow Yar1 to remain bound to the Rps3 N-domain until being removed by Ltv1. Moreover, this arrangement would also leave sufficient space for binding of Ltv1 to its interaction site at rRNA helix 41, and would orient the Rps3 N-domain towards rRNA helix 16, constituting the second Ltv1 crosslinking site^21^.

We conclude that while the Ltv1 N-domain, bound to the Rps3 N-domain, is likely positioned close to helix 16, the C-terminal part of Ltv1 (which also contains the Hrr25 phosphorylation site) interacts with Rps20 and is oriented towards helix 41 (Fig. 7c,d).

## Discussion

**Figure 8:**
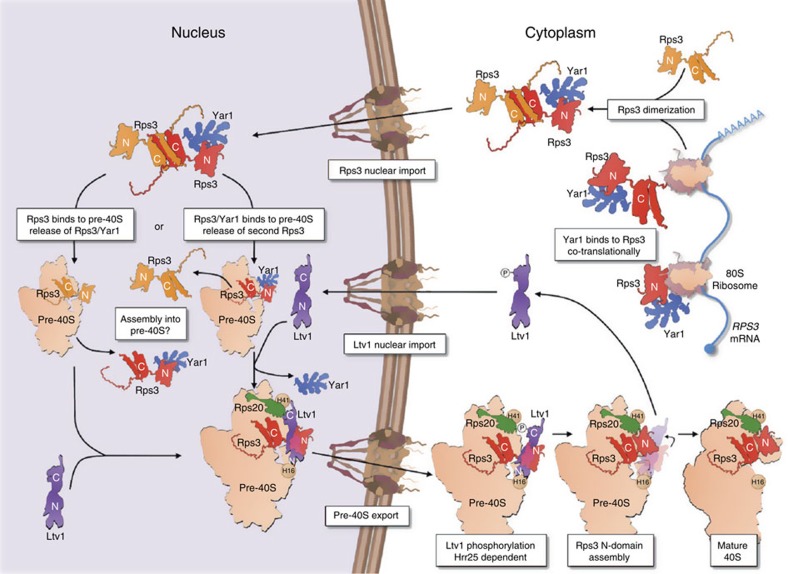
Model of the assembly path of Rps3. See Discussion for details. Note that the released, second Yar1-bound or free Rps3 may be incorporated into another pre-40S subunit (not depicted).

In this study, we have uncovered an intriguing mechanism involving a peculiar dimeric and rotated structure of the r-protein Rps3, and provided functional insights into the events underlying the stable integration of Rps3 during the final structural reorganization within the 40S beak region. On the basis of our data, we suggest a model for the assembly path of Rps3, which is shown in Fig. 8 and discussed below.

Newly synthesized Rps3 is captured co-translationally by its chaperone Yar1 (ref. 33), which protects the N-terminal part of Rps3. Following Rps3 synthesis, its C-domain dimerizes with a second Rps3 molecule by domain swapping. Our *in vitro* data suggest that the structural properties of the complex allow binding of two Yar1 molecules (one to each Rps3 N-domain); however, *in vivo*, only one Yar1 is present in this complex. This suggests that *in vivo* a mechanism exists that either prevents a second Yar1 from binding or that removes one Yar1 molecule quickly after formation of the tetrameric Rps3/Yar1 complex. Considering that Rps3 harbours an NLS in its N-domain (KKRK-motif, amino acids 7–10 (ref. 22)) directly adjacent to the Yar1-binding site (amino acids 11–30; Fig. 3a, left panel), it is unlikely that Yar1 and an importin can bind this region at the same time. It is tempting to speculate that importin binding may release one Yar1 molecule from the Rps3 dimer.

Another function of Rps3 dimerization might be protection from aggregation. Considering that Yar1 only protects the Rps3 N-domain but not the C-domain, which comprises the main rRNA-binding surface, dimerization likely serves to shield this part of the protein from unspecific interactions. In addition, the dimeric conformation might be cost-effective as it allows simultaneous targeting of two Rps3 molecules to ribosome assembly. Finally, the conformation of the Rps3/Rps3/Yar1 complex might be required to guide the incorporation of rotated Rps3 into pre-ribosomal particles.

Dissociation of the Rps3/Rps3/Yar1 complex occurs upon nuclear assembly of Rps3 into pre-ribosomal particles. Since domain swapping masks the major rRNA-binding site of the Rps3 C-domain, Rps3 dimers are presumably separated by competition with rRNA. It is not clear yet if a distinction is made whether the Yar1-bound Rps3 or the free Rps3 is incorporated. In our view, both events are possible—binding of Yar1-bound Rps3 would result in the subsequent release of Yar1 upon Ltv1 binding, while binding of free Rps3 would allow the direct binding of Ltv1. Once bound, Ltv1 prevents the final, salt-stable incorporation of the Rps3 N-domain in two ways: (1) the C-terminal part of Ltv1 interacts with Rps20 and the adjacent rRNA helix 41, hence occupying the final binding site of the Rps3 N-domain; and (2) the N-terminal part of Ltv1 binds to the Rps3 N-domain in proximity to rRNA helix 16, thereby fixing the Rps3 N-domain in its rotated conformation. We postulate that, in the cytoplasm, Ltv1 release is accomplished by a two-step mechanism: Hrr25-dependent phosphorylation of Ltv1 at serines 336, 339 and 342 leads to an electrostatic repulsion of the C-terminal part of Ltv1 from its rRNA-binding site at helix 41 and/or from Rps20, thereby making the site available for Rps3 binding. This event would loosen the ‘Ltv1 clamp' and relax the fixed conformation of Rps3, thus allowing a rotational movement of the Rps3 N-domain into its final assembly position where it interacts with Rps20 and rRNA helix 41. The force generated during the flip of the Rps3 N-domain may finally pull the Rps3-bound N-terminal part of Ltv1 off the second rRNA-binding site at helix 16, thereby leading to Ltv1 release.

Several bacterial r-proteins form their interactions with different rRNA-binding sites in sequential stages rather than in one step^34^. An example is r-protein S4, which initially only binds to part of its rRNA-binding site and induces folding events in the rRNA, which then results in the formation of additional interactions with the rRNA^35^.

It was observed that association of several eukaryotic r-proteins, including Rps3, with early pre-ribosomal particles is sensitive to salt treatment, and that salt stability of binding of these proteins increases with the progression of ribosome assembly^8^,^25^,^36^. Consequently, it was proposed that also eukaryotic r-protein assembly occurs stepwise, with only weak initial association to rRNA that is strengthened upon progression of ribosome maturation; however, it was not clear how such a transition might be achieved^8^,^25^,^36^. This study provides mechanistic insights into how an r-protein, which is initially attached loosely via only one domain, reaches its mature and stable conformation upon incorporation of the second interaction domain. Moreover, the direct coupling of r-protein assembly with the release of an assembly factor might represent a proofreading mechanism ensuring that only properly assembled 40S precursors can undergo final maturation steps and gain access to the pool of translationally active subunits. Another r-protein, Asc1, was reported to form dimers while exerting its extra-ribosomal function in cell signalling^37^, and it remains open whether Asc1 dimerization also precedes its ribosome incorporation. Future studies will have to determine whether other r-proteins are also assembled by a stepwise integration of individual domains and whether dimerization, as observed here for Rps3, is also used in their assembly paths.

## Methods

### Strains and plasmids

*Saccharomyces cerevisiae* strains used in this study are listed in Supplementary Table 2, and were obtained by established recombination, mating and tetrad dissection procedures. Plasmids used in this study were created using standard recombinant DNA techniques and are listed in Supplementary Tables 3 and 4.

### SAXS data collection, analyses and modelling

SAXS data with direct injection of the sample were collected on beamline BM29 at European Synchrotron Radiation Facility (ESRF; Grenoble, France) and SAXS data with size exclusion separation before data collection^38^ were collected on beamline SWING at the SOLEIL synchrotron (Saint-Aubin, France).

For direct injection, protein samples were centrifuged at 13,200*g* for 10 min before data acquisition and concentration was measured with NanoDrop spectrophotometer (Thermo). The samples were then loaded at a flow rate of 200 μl min^−1^ in the SAXS capillary flow cell. Experiments on BM29 were done at an electron energy of 8 keV, and data were recorded on a Pilatus 1M detector (Dectris). Ten 1-s exposure frames were collected for each sample. For size exclusion chromatography, samples were loaded on an Agilent SEC-3 size-exclusion chromatography column connected to an Agilent high-performance liquid chromatography system at a flow rate of 200 μl min^−1^. Experiments on SWING were done at an electron energy of 13.32 keV, and data were recorded on PCCD170 detector (AVIEX). One hundred frames, with exposure times of 1 s per frame, corresponding to the elution peak were collected at 288 K in the flow cell connected to high-performance liquid chromatography system. The same buffer was used for all data collection (Tris-HCl 50 mM and NaCl 200 mM, pH 7.5). Buffer scattering was collected in the same conditions or using the gel filtration profile before the void volume.

Data reduction, image conversion to 1D profile, absorption correction, scaling to absolute scale, buffer subtraction, averaging and peak analysis were carried out using the pipeline available on BM29 beamline or using the program FOXTROT available on the SWING beamline. Further processing and data analysis was done using the programs of the ATSAS suite^39^. Guinier plot analysis was performed using PRIMUS with scattering data at low-angle regions with qmax × Rg<1.3. Pair distribution function was calculated with GNOM. *Ab initio* analysis was done with DAMMIF. In total, 50 DAMMIF calculations were averaged with DAMAVER to produce averaged and filtered shape. Homology models were made with MODELLER^40^.

Modelling with SAXS data was done with DADIMODO^41^ using the core structural domain from the crystal structure and fitting for flexible N- and C-terminal extensions. The N-terminal (1–29) and C-terminal (207–214) extensions of Yar1 (1–214 including tag) were made flexible. For Rps3 (1–249 including tag), N-terminal (1–16) and C-terminal (203–249) extensions were modelled flexible. Flexibility between the N- and C- domains (101–105) was introduced.

Analysis of the fit between model and experimental data was done with CRYSOL.

The reduced chi-square calculation for a data set containing *N* data points is defined as


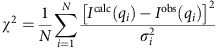


The relative residual is given by


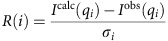


For free chi-square calculation^42^, the data set is divided in *n*_*S*_ bins, where *n*_*S*_ is the number of Shannon channels.


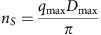


During a selection round *k*, a single data point is randomly chosen in each bin. *χ*^2^ is calculated with these *n*_*S*_ points.


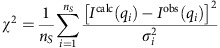


*χ*^2^_free_ is the median of the *χ*^2^ calculated over *k* rounds of selection. Typically, *k*=1,001.

The cumulative chi-square is given by


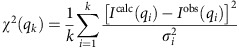


For chi-square calculation by momentum transfer bins, the data set is divided in *n*_*S*_ bins.

For a bin *k* (*k* from 1 to *n*_*S*_)


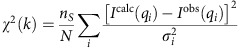


with 
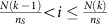


To model the full-length Rps/Yar1 complex, the following stepwise strategy was used:

We first used the SAXS data for Yar1 full-length protein (Yar1-FL) to model Yar1 alone. Model structures were generated with a rigid structural core corresponding to the residues observed in the crystal structure of the Yar1-Rps3 complex (Yar1(7–156)), and flexible N- and C-terminal extensions (29 N-terminal residues including 14 residues from the His-tag and 55 C-terminal residues). The conformations of the flexible extensions were optimized for a best fit against the experimental data, and were fitted into the independently determined molecular envelope. The models showed a good agreement with the experimental data with an overall *χ*^2^ of 1.06 (*χ*=1.03, *χ*^2^free=1.04).

We next used SAXS data for the complex between the Yar1 structural core lacking the N- and C-terminal extensions (Yar1(8–153)) and the Rps3 N-domain. The model was created by using the same conformations for the Yar1 core and the Rps3 N-domain as in the crystal structure. The N- and C-terminal residues of each construct that are missing in the crystal structure were built and refined against the experimental data. This includes the 14 N-terminal residues of the Rps3 N-domain. The orientation of the two molecules was not fixed in the calculations, allowing for variations in the overall structure of the complex. The obtained model showed a good agreement with the experimental data (*χ*^2^=1.056, *χ*=1.028 and *χ*^2^free=0.996).

We then used the data with the Rps3 N-domain in complex with Yar1-FL. Although we do not observe any interaction-induced folding of the Yar1 extensions, they are in a slightly more compact conformation than in the free Yar1, as evidenced by the smaller radius of gyration (28.6 Å). By using the models from the determined structures of Rps3 N-domain/Yar1-core and isolated Yar1-FL, an initial model was built and refined for all the unstructured regions. The refined model shows a good agreement with the experimental data (*χ*^2^=1.380, *χ*=1.175 and *χ*^2^free=1.306). The core domains of the complex stay in the same conformation as in the crystal structure, and the unstructured regions adopt an unfolded and extended conformation.

Finally, for the model of the dimeric full-length Rps3/Yar1 complex, initial models of the three possible dimers were built from the crystal structures, and completed with the unstructured regions determined in the SAXS model of the Rps3 N-domain/Yar1 complex. All the extensions and relative molecule orientations were then optimized against the experimental data.

### Purification of His6-tagged proteins

Expression constructs were cloned into a pETDuet-1 vector (Novagen) and transformed in an *Escherichia coli* BL21 (DE3) Rosetta Star strain. Cells were cultured in LB medium at 30 °C to an OD_600_ of 0.3–0.4. Protein expression was induced with 0.3 mM isopropyl β-D-thiogalactoside (IPTG), and cultures were shifted to 16 °C for 20 h. Cell pellets were resuspended in lysis buffer containing 50 mM Tris (pH 7.5), 150 mM NaCl, 40 mM imidazole, HP protease inhibitor mix (Serva), 0.5 mM phenylmethylsulphonyl fluoride (PMSF), 1 mM dithiothreitol (DTT) and 1 mg ml^−1^ lysozyme, and lysed by sonication. Cell lysates were centrifuged at 40,000*g* at 4 °C for 30 min to remove insoluble material, and supernatants were incubated for 1 h under rotation at 4 °C with Ni-NTA agarose (Qiagen). Beads were then washed three times with ∼10 ml washing buffer (50 mM Tris (pH 7.5), 150 mM NaCl, 40 mM imidazole and 1 mM DTT), and bound material was subsequently eluted under rotation for 20 min at 4 °C with 300 mM imidazole. When indicated in the respective figures, the obtained eluates were subjected to a second affinity purification step to pull down co-purified interaction partners with anti-Flag agarose (Sigma). After incubation for 1 h under rotation at 4 °C, beads were washed four times with washing buffer (50 mM Tris (pH 7.5), 150 mM NaCl and 1 mM DTT), and bound material was eluted with 100 μg ml^−1^ Flag-peptide (Sigma). Eluates were analysed by SDS–polyacrylamide gel electrophoresis (SDS–PAGE) using 12% or 4–12% polyacrylamide gels (Invitrogen), and protein bands were visualized by Coomassie staining or western blotting.

### Purification of Flag-tagged proteins

Flag-Yar1 was expressed from the pETDuet-1 vector (Novagen), Ltv1-Flag and Enp1-Flag-TEV-ProteinA (FPA) were expressed from the pET15b vector (Novagen) with the conditions described above. Cell pellets were resuspended in lysis buffer containing 50 mM Tris (pH 7.5), 150 mM NaCl, HP protease inhibitor mix (Serva), 0.5 mM PMSF, 1 mM DTT and 1 mg ml^−1^ lysozyme, and lysed by sonication. Cell lysates were centrifuged at 40,000*g* and 4 °C for 30 min to remove insoluble material, and supernatants were incubated for 1 h under rotation at 4 °C with anti-Flag agarose (Sigma). Beads were then washed three times with ∼10 ml washing buffer (50 mM Tris (pH 7.5), 150 mM NaCl and 1 mM DTT), and bound material was subsequently eluted under rotation for 45 min at 4 °C with 100 μg ml^−1^ Flag-peptide (Sigma).

For the experiment in Fig. 7a, Flag-tagged Ltv1 truncations were co-expressed with His6-Rps20 from the pETDuet-1 vector and affinity-purified with anti-Flag agarose as described above. Lysis and washing buffers contained 400 mM NaCl.

### Purification of GST-TEV-Hrr25

GST-TEV-Hrr25 was expressed from the pProEx-1 vector^43^ after induction with 0.3 mM IPTG for 16 h at 18 °C. Cell pellets were resuspended in lysis buffer containing 50 mM Tris (pH 7.5), 150 mM NaCl, HP protease inhibitor mix (Serva), 0.5 mM PMSF, 1 mM DTT and 1 mg ml^−1^ lysozyme, and lysed by sonication. Cell lysates were centrifuged at 40,000*g* and 4 °C for 30 min to remove insoluble material, and supernatants were incubated for 1 h under rotation at 4 °C with glutathione (GSH) agarose (Sigma). Beads were then washed three times with ∼10 ml washing buffer (50 mM Tris (pH 7.5), 150 mM NaCl and 1 mM DTT), and bound GST-TEV-Hrr25 was eluted under rotation for 1 h at room temperature with tobacco etch virus (TEV) protease.

### *In vitro* phosphorylation assay

Affinity-purified Ltv1-Flag, Ltv1(S336A/S339A/S342A)-Flag and Ltv1(S6A)-Flag were incubated with affinity-purified Hrr25 for 20 min at 30 °C in a buffer containing 50 mM Tris (pH 7.5), 150 mM NaCl, 5 mM MgCl_2_, 0.1 mM ATP and 1 mM DTT. Reactions were stopped by addition of SDS–PAGE loading buffer and boiling at 95 °C for 10 min. Samples were analysed on 4–12% SDS–polyacrylamide gels and Coomassie staining or western blotting with biotin-labelled Phos-tag (Wako Pure Chemical Industries) in combination with streptavidin-horseradish peroxidase (Life Technologies).

### Tandem affinity purification

TAP purifications were performed as described previously^44^. Diploid strains used for experiments shown in Fig. 2 were grown at 25 °C, strains used for purifications shown in Supplementary Fig. 4a were grown at 30 °C. Cell pellets were resuspended in lysis buffer containing 50 mM Tris (pH 7.5), 100 mM NaCl, 2 mM MgCl_2_, 0.075% NP-40, 1 mM DTT, 0.5 mM PMSF and 1 × FY protease inhibitor mix (Serva), and lysed by mechanical disruption with glass beads. Lysates were cleared by centrifugation, mixed with IgG Sepharose 6 Fast Flow (GE Healthcare) and incubated at 4 °C for 90 min. Beads were spinned down, washed with lysis buffer (without protease inhibitors) and transferred into Mobicol columns (MoBiTec). After washing with 20 column volumes, samples were eluted with TEV protease for 75 min at RT. After addition of 2 mM CaCl_2_, eluates were incubated with Calmodulin Sepharose 4B (GE Healthcare) for 1 h at room temperature. Beads were washed with 10 column volumes of lysis buffer containing 2 mM CaCl_2_ and eluted with buffer containing 5 mM EGTA. For the experiments shown in Fig. 2, the obtained EGTA eluates were incubated with anti-Flag agarose (Sigma) for 1 h at 4 °C. Beads were washed with eight column volumes of lysis buffer, and bound proteins were subsequently eluted with 100 μg ml^−1^ Flag-peptide (Sigma). Final eluates were precipitated, resuspended in SDS sample buffer and loaded onto 12% (Fig. 2b,d) or 4–12% (Supplementary Fig. 4a) NuPAGE Bis-Tris gels (Thermo Fisher).

Uncropped scans of Coomassie-stained gels and western blots in Fig. 2b,d are shown in Supplementary Fig. 8.

### Sucrose gradient analysis

Cells were grown at 30 °C in 100 ml yeast extract peptone dextrose (YPD) medium to logarithmic growth phase (*A*_600_ of ∼0.6). Approximately, 100 μg ml^−1^ CHX was added to the cultures and after incubation for 5 min on ice, cells were pelleted and resuspended in lysis buffer (10 mM Tris-HCl (pH 7.5), 100 mM NaCl, 30 mM MgCl_2_ and 100 μg ml^−1^ CHX). After cell lysis with glass beads, 5 *A*_260_ units of the cell extracts were loaded onto 7–45% sucrose gradients and centrifuged at 180,000*g* for 2 h 45 min at 4 °C. Gradients were analysed using a UA-6 system (Teledyne Isco) with continuous monitoring at *A*_254 nm_.

### Western blotting

Western blot analysis was performed using the following antibodies: anti-Yar1 antibody (1:5,000; ref. 22^22^), anti-Rps3 antibody (1:30,000, provided by Matthias Seedorf), anti-Rps8 antibody (1:5,000, provided by Giorgio Dieci), anti-Rps2/Rpl30 antibody (1:2,000, provided by Jonathan Warner), anti-hRps5 antibody (1:500, Sigma-Aldrich, cat. no. HPA055878), anti-Rpl35 antibody (1:35,000, provided by Matthias Seedorf), anti-Rps26/Tsr2 antibody (1:2,000, provided by Vikram Panse), anti-Ltv1 antibody (1:8,000, provided by Katrin Karbstein), anti-Enp1 antibody (1:4,000, provided by Katrin Karbstein), anti-Tsr1 antibody (1:4,000, provided by Katrin Karbstein), anti-Dim1 antibody (1:4,000, provided by Katrin Karbstein), anti-Rio2-antibody (1:500, Santa Cruz Biotechnology, cat. no. sc-98828), anti-CBP antibody (1:4,000, Merck-Millipore, cat. no. 07-482), secondary anti-rabbit horseradish peroxidase-conjugated antibody (1:15,000, Sigma-Aldrich, cat. no. A0545), horseradish peroxidase-conjugated anti-His6 antibody (1:15,000, Sigma-Aldrich, cat. no. A7058), horseradish peroxidase-conjugated anti-Flag antibody (1:15,000, Sigma-Aldrich, cat. no. A8592).

The biotinylated phosphate-binding reagent Phos-Tag (Wako Pure Chemical Industries) was prepared according to manufacturer's instructions and used to detect protein phosphorylation in combination with streptavidin-HRP (Invitrogen).

### Y2H interaction analyses

Plasmids expressing the bait proteins, fused to the GAL4 DNA-binding domain (G4BD), and the prey proteins, fused to the GAL4 activation domain (G4AD), were co-transformed into the reporter strain PJ69-4A. Y2H interactions were documented by spotting representative transformants in 10-fold serial dilution steps on synthetic dextrose complete (SDC)-Trp-Leu, SDC-Trp-Leu-His (HIS3 reporter) and SDC-Trp-Leu-Ade (ADE2 reporter) plates. Growth on SDC-Trp-Leu-His plates is indicative of a weak interaction, whereas only relatively strong interactions permit growth on SDC-Trp-Leu-Ade plates.

### RNA isolation and northern blotting

Total RNA preparations were performed from 20 *A*_600_ units using the mechanical disruption protocol of the RNeasy mini kit (Qiagen). Approximately, 3 μg of RNA per sample were separated on 1.5% MOPS-agarose gels as described in the manual for the RNeasy mini kit. The RNA was transferred overnight onto a Hybond-N nylon membrane (Amersham Biosciences), and then crosslinked to the membrane by ultraviolet radiation. Hybridization was performed overnight at 42 °C in 500 mM NaPO_4_ buffer (pH 7.2), 7% SDS and 1 mM EDTA using 5′-^32^P-labelled oligonucleotides with the following sequences: 20S, 5′-GACTCTCCATCTCTTGTCTTCTTG-3′; 25S, 5′-CTCCGCTTATTGATATGC-3′; 18S, 5′-CATGGCTTAATCTTTGAGAC-3′. The membranes were washed three times for 20 min at 42 °C in 40 mM NaPO_4_ buffer (pH 7.2) and 1% SDS, and radioactivity was detected by exposing X-ray films. Membranes were regenerated by washing in 1% SDS.

### Fluorescence microscopy

Life yeast cells were imaged by fluorescence microscopy using a Zeiss Axioskop microscope. When indicated, 0.2 μg ml^−1^ LMB and 20 μg ml^−1^ CHX were added.

## Additional information

**Accession codes:** SAXS data have been deposited in the BIOISIS depository with the IDs YAR1P (Yar1 full-length protein), Y1R3DP (Yar1 core/Rps3 N-domain), YRNTRP (Yar1/Rps3 N-domain) and YFLRNP (Yar1/Rps3).

**How to cite this article:** Mitterer, V. *et al.* Sequential domain assembly of ribosomal protein S3 drives 40S subunit maturation. *Nat. Commun.* 7:10336 doi: 10.1038/ncomms10336 (2016).

## Supplementary Material

Supplementary Information Supplementary Figures 1-8, Supplementary Tables 1-4 and Supplementary References
