# Supplementary material for: Sequential domain assembly of ribosomal protein S3 drives 40S subunit maturation
Source: Nat Commun. 2016 Feb 2;7:10336. doi: 10.1038/ncomms10336 (PMC4740875; doi:10.1038/ncomms10336)
Supplement: Supplementary Information — Supplementary Figures 1-8, Supplementary Tables 1-4 and Supplementary References [file ncomms10336-s1.pdf]

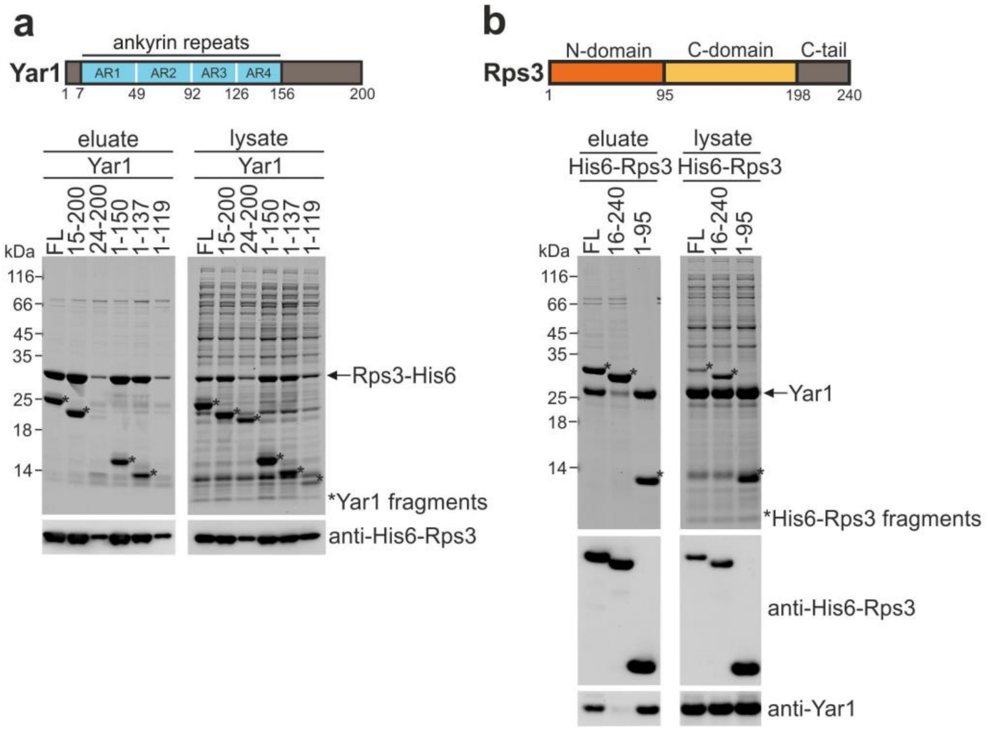

Supplementary Figure 1. Yar1 ankyrin-repeat core interacts with the Rps3 N-domain

(a) The Yar1 ankyrin-repeat core (~amino acids 15-137) interacts with Rps3 in vitro. His6-Rps3 and the indicated Yar1 truncations were co-expressed in *E. coli* and after cell lysis purified via Ni-NTA agarose beads. Cell lysates and Ni-NTA eluates were analyzed by SDS-PAGE and Coomassie staining or western blotting. Note that upon co-expression of Rps3 with non-interacting Yar1 fragments (i.e. fragments 24-200 and 1-119) the r-protein becomes insoluble resulting in decreased His6-Rps3 yields in the respective eluates. Asterisks indicate the different Yar1 fragments. **(b)** Rps3 N-domain is necessary and sufficient for interaction with Yar1. His6-tagged full-length Rps3 (FL), an Rps3 fragment lacking the N-terminal 15 amino acids (16-240) or the Rps3 N-domain (1-95) were co-expressed with full-length Yar1 in *E. coli.* After cell lysis, the His6-tagged proteins were purified via Ni-NTA agarose. Cell lysates and Ni-NTA eluates were analyzed by SDS-PAGE and Coomassie staining or western blotting. Asterisks indicate the different Rps3 fragments.

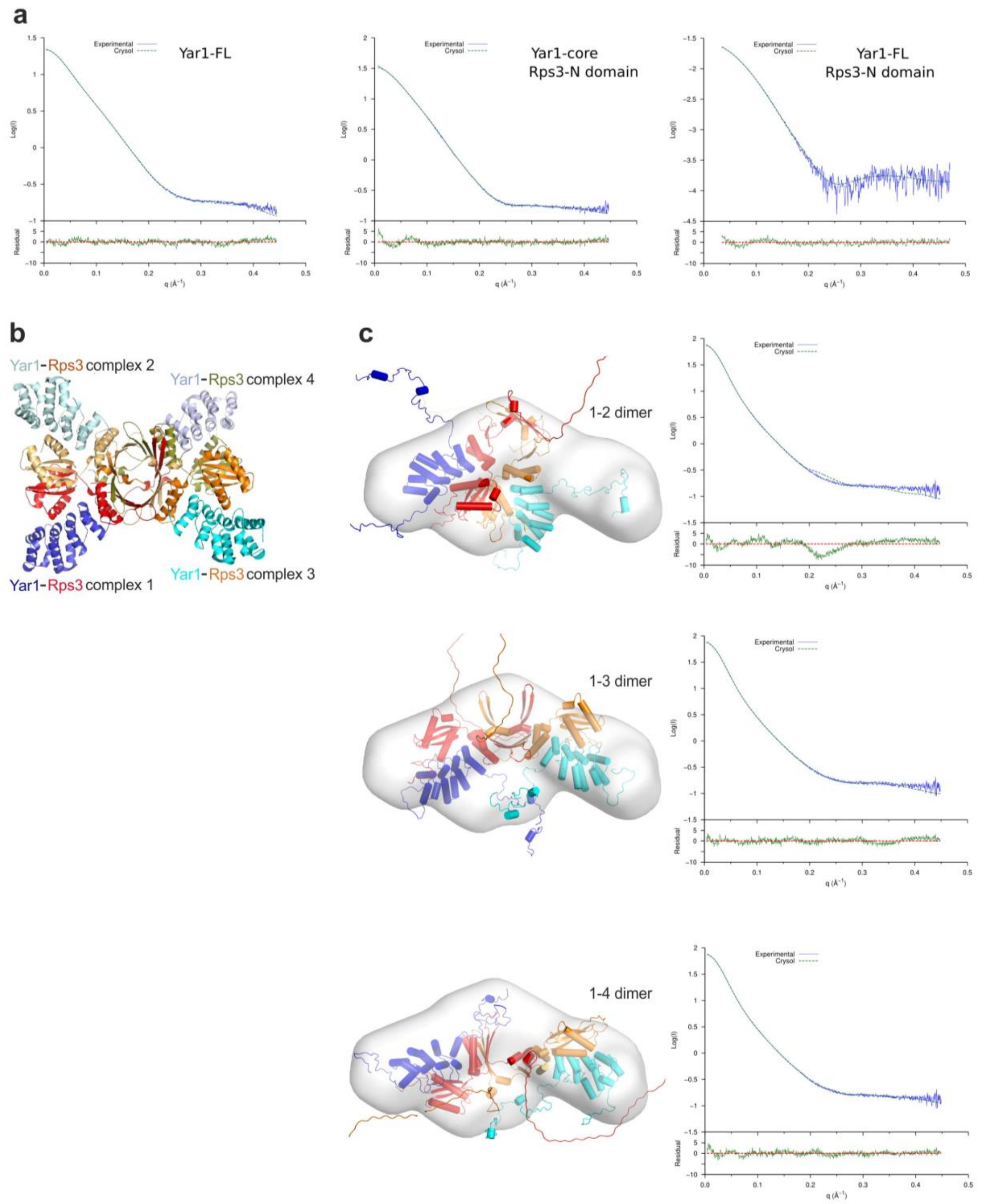

Supplementary Figure 2. SAXS of Rps3/Yar1 full-length complex and truncations

(a) SAXS scattering curves of Rps3 N-domain/Yar1 complexes. Experimental (blue line) and calculated (dashed green line) scattering curves for the complexes shown in **Figure 1a**. **(b)** Definition of the dimers present in the Rps3/Yar1 crystal structure. The Rps3/Yar1 complexes are numbered and color-coded as indicated in the labels. Each complex contacts

three other complexes, resulting in three possible dimer conformations (e.g. complex 1 contacts complexes 2, 3, and 4, resulting in dimers 1-2, 1-3 and 1-4). **(c)** SAXS modeling of full-length Rps3/Yar1 complexes. Models (left) and fit to the experimental (blue line) and calculated (dashed green line) scattering curves (right) of Rps3/Yar1 dimers, for the three possible conformations extracted from the crystal structure in **b**. All models were fit into the same representative envelope. Yar1 is colored blue and light blue, Rps3 is colored red and orange. The best fit was observed with dimer 1-4. The model of dimer 1-4 is also shown in **Figure 1b**.

a

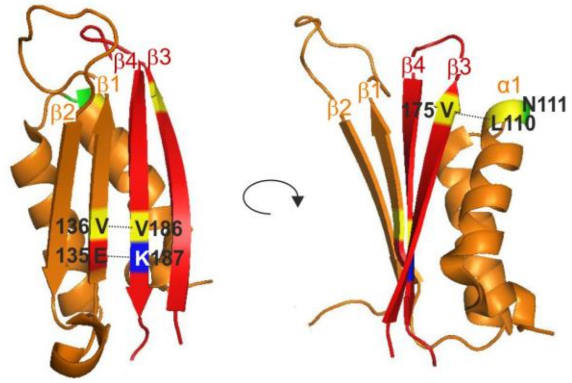

b

dimer contacts	mutant	exchanges
E135-K187	SL1	T46A R64K **E135G**
	SL3	E28D **K187E**
V136-V186	SL5	T70A **V186A**
L110-V175	SL2	F25S **L110S** K223R
	SL4	I84T **N111Y**

Supplementary Figure 3. *rps3* mutants displaying synthetic growth defects with the *yar1*Δ mutant contain mutations in residues implicated in domain swapping contacts

(a) Regions of Rps3 involved in domain swapping are shown in two orientations (structure extracted from **Figure 1d**, left panel). Elements belonging to the two different Rps3 protomers are shown in red and orange, respectively. Amino acids involved in important dimer contacts are indicated. **(b)** *rps3* mutants isolated in a screen for enhanced growth defects in combination with *yar1* deletion (see also[1]). The exchanges in the respective mutants are indicated. Exchanges affecting the contacts involved in domain swapping are highlighted in bold. The fifth mutant isolated in the screen (SL4) contained an N111Y exchange. N111 is not in direct contact with the domain swapped β-sheets, however, mutation of N111 likely results in an altered positioning of L110, which might disrupt the interaction between L110 and V175.

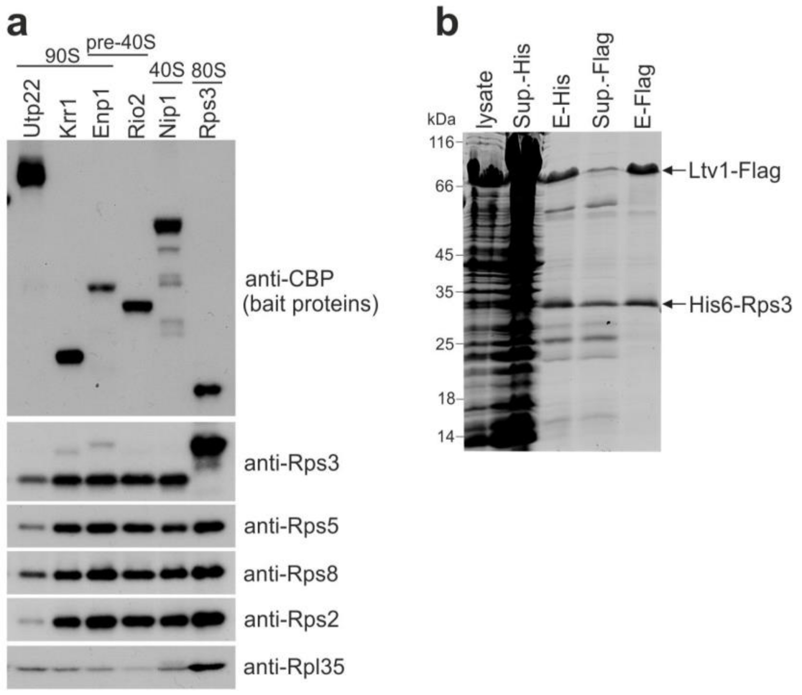

Supplementary Figure 4. Rps3 is assembled into pre-ribosomes as a monomer

(a) Amounts of Rps3 relative to other r-proteins are equal in pre-ribosomal particles and mature 40S subunits. TAP-eluates of the indicated bait proteins were analyzed by SDS-PAGE and western blotting with the indicated antibodies against r-proteins. The large subunit r-protein Rpl35 was detected to estimate the extent of contamination of the purifications with mature ribosomes and is specifically co-purified only in the Rps3 TAP-purification. **(b)** Rps3 and Ltv1 directly interact in vitro. His6-Rps3 and Ltv1-Flag were co-expressed in *E. coli*. In a first purification step, His6-Rps3 was pulled down with Ni-NTA agarose and the obtained eluate (E-His) was subjected to a second purification step with anti-Flag agarose (E-Flag). Cell lysate, unbound supernatants of the two purifications steps (Sup.-His, Sup.-Flag) and the eluates (E-His, E-Flag) were analyzed by SDS-PAGE and Coomassie staining.

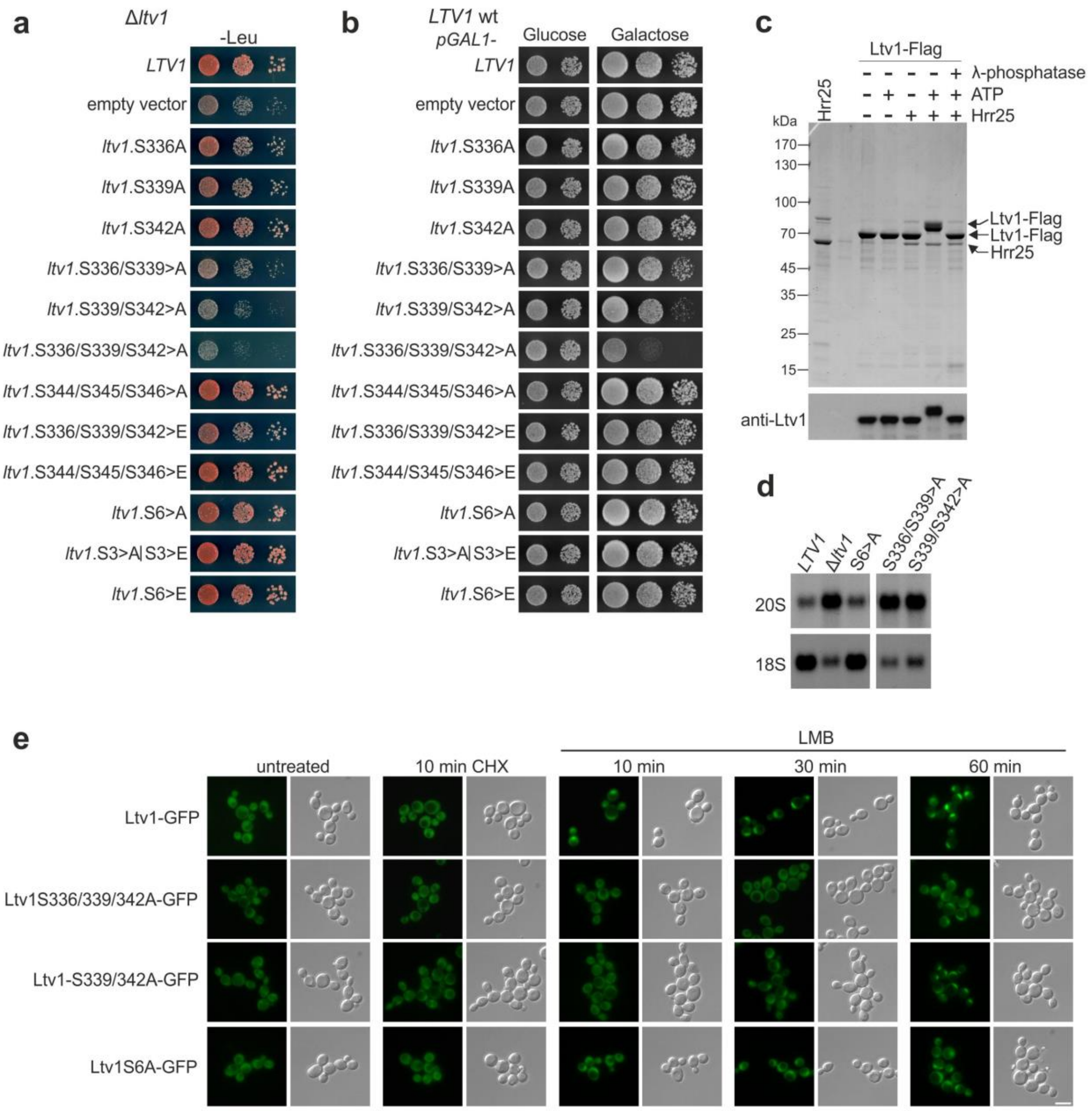

Supplementary Figure 5. Serines 336, 339, and 342 comprise the main Ltv1 phosphorylation site

(a) Growth phenotype of *ltv1* mutants. The growth phenotypes of the indicated *ltv1* mutant strains were assessed on SD-Leu plates incubated at 23°C for 3 d. *ltv1*.S6A and *ltv1*.S6E: all six serines (see **Fig. 4a**) were mutated to alanine and glutamate, respectively. *ltv1*.S3A/S3E: the first three serines were mutated to alanine, the last three serines to glutamate. **(b)** Dominant negative effects upon overexpression of *ltv1* phosphomutants. Wild-type *LTV1* and

indicated *ltv1* mutants were overexpressed from the galactose inducible *GAL1-10* promoter in wild-type cells. The growth was examined after incubation on glucose (repressed condition) or galactose (induced condition) containing plates at 23°C for 3 and 7 d respectively. **(c)** Ltv1 is efficiently phosphorylated by Hrr25 in vitro. Ltv1-Flag and GST-TEV-Hrr25 were expressed in *E. coli* and purified with anti-Flag agarose and elution with Flag-peptide or GSH agarose and elution with TEV protease, respectively. The purified proteins were incubated in the presence or absence (+/-) of ATP. In addition, λ-phosphatase was added to one sample additionally to ATP and Hrr25. Samples were analyzed by SDS-PAGE and Coomassie staining or Western blotting. The efficient Ltv1 phosphorylation is obvious from the full bandshift to a slower migrating band in the gel. **(d)** rRNA processing defects in *ltv1* phosphomutants. The indicated *ltv1* mutants were analyzed by Northern blotting with probes directed against immature 20S and mature 18S rRNA species. In contrast to the other *ltv1* phosphomutants, 20S processing was not affected in the Ltv1(S6A) mutant. **(e)** Delayed nuclear recycling of phosphomutated Ltv1. The localization of wild-type Ltv1 or of the indicated phosphomutant proteins fused to GFP was examined in leptomycin B (LMB) sensitive *crm1* mutant cells prior to and after cycloheximide (CHX) and LMB treatment. LMB was added after incubating the cells for 10 min with CHX. Note that, while the Ltv1(S336A/S339A)- and Ltv1(S336A/S339A/S342A)-GFP fusions both displayed a delay in recycling back to the nucleus, the nuclear trapping of the Ltv1(S6A)-GFP occurred with an efficiency comparable to wild-type Ltv1. Scale bar is 5 µm.

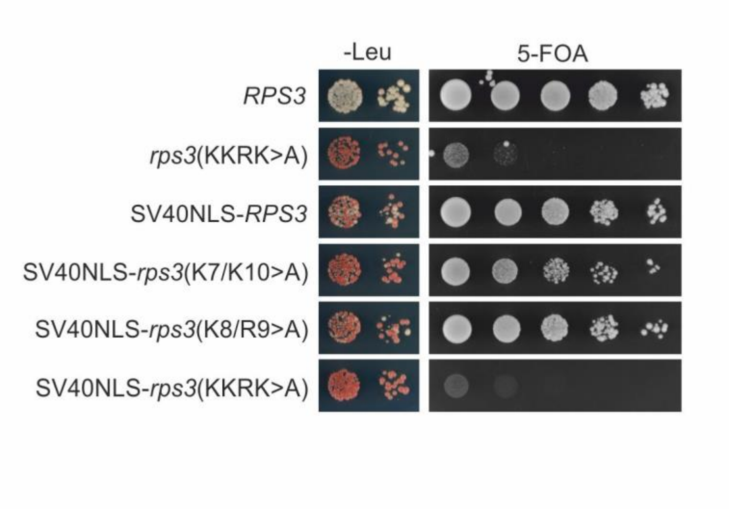

Supplementary Figure 6. SV40-NLS fusion does not restore growth defects of *rps3*-(KKRK>A) mutants

An *rps3*Δ strain supplemented with *RPS3* on a *URA3*-plasmid was transformed with plasmids carrying wild-type *RPS3*, the *rps3*(KKRK>A) mutant or the indicated *rps3* alleles fused with the nuclear localization signal (NLS) of the SV40 large T-antigen. The growth phenotypes after loss of the *URA3-RPS3* wild-type copy were examined on 5-FOA containing plates incubated at 23°C for 4 d.

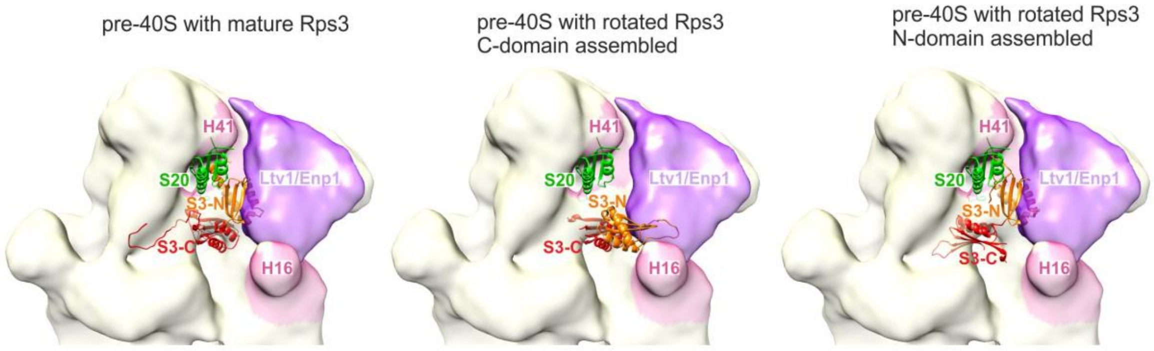

Supplementary Figure 7. Model of Rps3 assembled to pre-40S particles

Cryo-EM density of the *S. cerevisiae* pre-40S ribosomal particle isolated from *ltv1*Δ cells (Rio2-TAP; emdb 1924, shown in white); including the difference density derived from the corresponding wild-type pre-40S particle (emdb 1927), represented in violet and most likely containing Ltv1 and Enp1[2]. The rRNA interaction sites of Ltv1 identified by UV-crosslinking and cDNA analysis (CRAC)[3], comprising helix 16 and helix 41, are displayed in pink. Left panel: Rps20 (PDB 3U5C) and Rps3 (PDB 3U5C) were placed by modeling the mature 40S structure (PDB 3U5C) into the cryo-EM density of the pre-40S particle. Middle and right panel: Rps20 (PDB 3U5C; shown in green) and rotated Rps3 from the Rps3/Yar1 complex (PDB 4BSZ) were modeled into the cryo-EM structure, assuming that Rps3 is assembled via the Rps3 C-domain (shown in red; middle panel) or the Rps3 N-domain (shown in orange; right panel). Note that in the mature and N-domain assembled orientation, the Rps3 N-domain is in contact with helix 41, while in the rotated conformation with the C-domain assembled, the N-domain comes close to helix 16. The Figure was generated with Chimera[4].

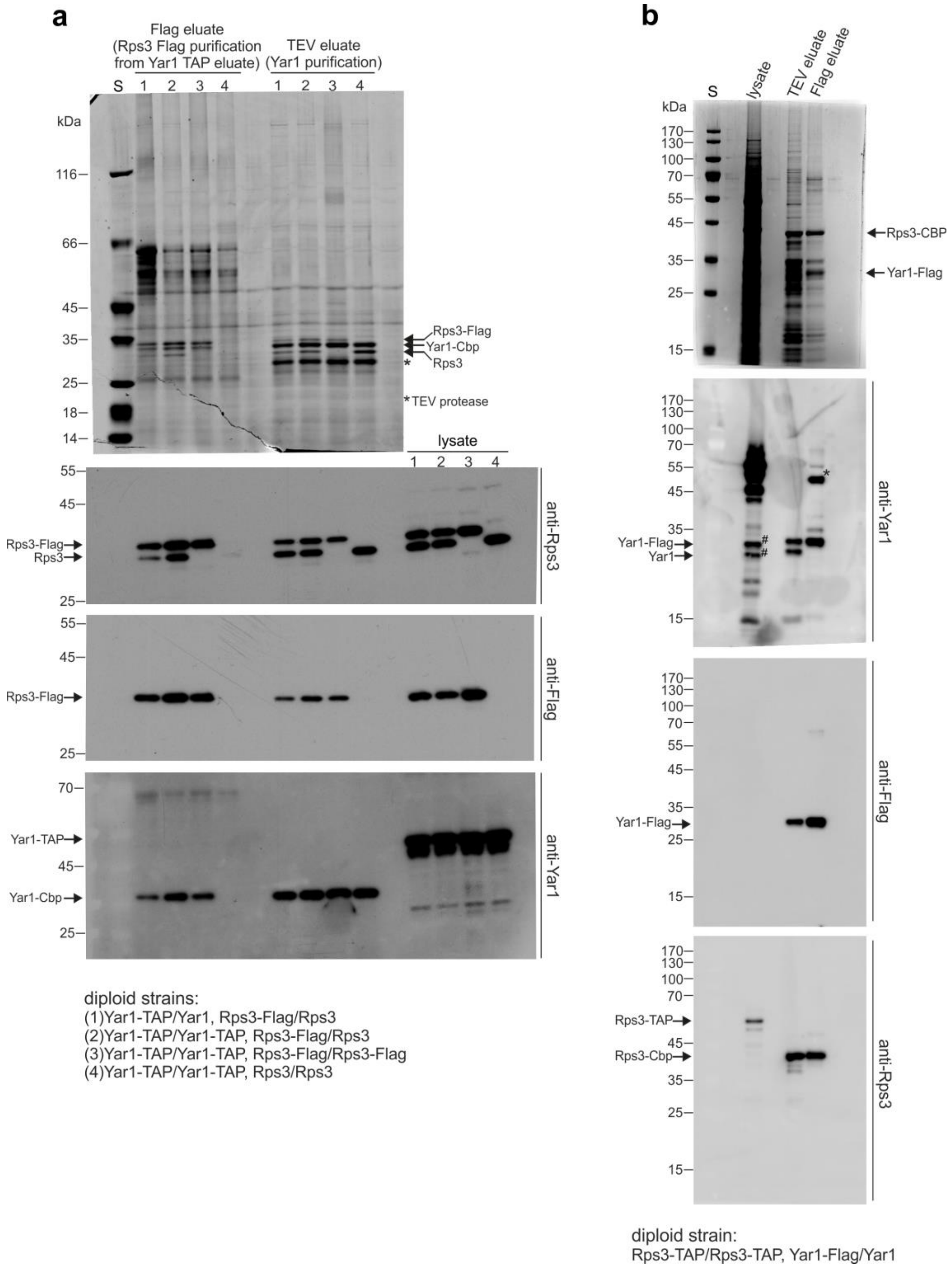

Supplementary Figure 8. Uncropped scans of Coomassie stained gels and western blots shown in Figures 2b and 2d. (a) The strong Yar1-TAP signal in the anti-Yar1 western blot results from binding of proteinA to the secondary peroxidase-conjugated antibody. **(b)** The band marked with an asterisk (*) in the Yar1 western blot might represent traces of contamination with uncleaved Rps3-TAP protein and appear due to binding of proteinA to the

secondary peroxidase-conjugated antibody. Hashes indicate degradation products of Rps3-TAP. S=protein standard

Supplementary Table 1. SAXS data collection and modeling statistics

	Yar1 FL	Yar1 core/ Rps3 N-domain	Yar1 FL/ Rps3 N-domain	Yar1 FL/ Rps3 FL
**Data-collection parameters**				
Instrument	SWING	SWING	SWING	SWING
Beam geometry (mm)	0.4×0.1	0.4×0.1	0.4×0.1	0.4×0.1
Wavelength (Å)	1.03	1.03	1.03	1.03
q range ($Å^{-1}$)	0.07-0.5	0.07-0.5	0.07-0.5	0.07-0.5
Exposure time (s) / nb frames	1 / 100	1 / 100	1 / 100	1 / 100
Concentration range ($mg.ml^{-1}$)	2-10	2-10	2-10	2-10
Temperature	288	288	288	288
**Structural parameters**				
I(0) (cm^{-1}) [from P(r)]	0.24	0.27	0.29	0.61
Rg (Å) [from P(r)]	32.5	21.4	28.6	45.5
I(0) (cm^{-1}) [from Guinier]	0.23	0.27	0.29	0.61
Rg (Å) [from Guinier]	32.6	21.5	28.5	45.7
D_{max} (Å)	114.2	74.9	100	155.7
Porod estimate ($Å^3$)	44004	45369	54977	183758
Dry volume calculated from sequence ($Å^3$)	29000	34150	44037	62408
**Molecular-mass determination**				
Partial specific volume ($cm^3.g^{-1}$)	0.707	0.720	0.718	0.728
Contrast ($\Delta\rho \times 10^{10}$ cm^{-2})	3.259	2.980	3.074	2.930
Molecular mass M_r [from I(0)]	27502	28355	34250	120851
Calculated monomeric M_r from sequence	23944	28224	36376	51559
**Software employed**				

Primary data reduction	FOXTROT
Data processing	PRIMUS
Ab initio analysis	DAMMIF
Validation and averaging	DAMAVER
Rigid-body modeling	DADIMODO
Computation of model intensities	CRYSOL
Three dimensional graphics representation	PyMOL

Supplementary Table 2. Yeast strains

Name	Genotype	Source
W303	*ade2-1, his3-11, 15, leu2-3,112, trp1-1, ura3-1, can1-100*	Thomas and Rothstein, 1989
C303	*ADE2* wild-type integration into W303	this study
YDK11-5A	W303 *MAT*α *ade3*::kanMX4	Kressler et al., 1999
*ltv1*Δ (YGM31)	W303 *MAT*α *ltv1*::*HIS3*MX4	this study
*hrr25*Δ shuffle *ltv1*Δ (YGM263)	W303 *MAT*α *hrr25*::natNT2 *ltv1*::*HIS3*MX4 *ade3*::kanMX4 [pHT4467Δ-*HRR25*]	this study
*rps3*Δ shuffle (YGM90)	W303 *MAT*α *rps3*::natNT2 *ade3*::kanMX4 [pHT4467Δ-*RPS3*]	this study
*rps3*Δ shuffle *ltv1*Δ (YGM89)	W303 *MAT***a** *rps3*::natNT2 *ltv1*::*HIS3*MX4 *ade3*::kanMX4 [pHT4467Δ-*RPS3*]	this study
*rps3*Δ shuffle *yar1*Δ (YGM187)	W303 *MAT*α *rps3*::natNT2 *yar1*::*HIS3*MX4 *ade3*::kanMX4 [pHT4467Δ-RPS3]	this study
*rps20*Δ shuffle (YGM286)	W303 *MAT***a** *rps20*::natNT2 *ade3*::kanMX4 pHT4467Δ-*RPS20*	this study
*rps20*Δ shuffle *ltv1*Δ	W303 *MAT***a** *rps20*::natNT2 *ltv1*::hphNT1*ade3*::kanMX4 [pHT4467Δ-*RPS20*]	this study
*rps20*Δ shuffle *ltv1*Δ (YGM301)	W303 *MAT***a** *rps20*::natNT2 *ltv1*::*HIS3*MX4 *ade3*::kanMX4 [pHT4467Δ-*RPS20*]	this study
*rps3*Δ shuffle *rps20*Δ shuffle (YGM295)	W303 *MAT*α *rps3*::natNT2 *rps20*::*HIS3*MX4 *ade3*::kanMX4 [pHT4467Δ-*RPS3*] [YCplac33-*RPS20*]	this study
*crm1-1 ltv1*Δ	C303 *MAT***a** *crm1*T539C::kanMX4 *ltv1*::*HIS3*MX6	this study
UTP22-TAP	W303 *MAT***a** *UTP22*-TAP::natNT2	Koch et al., 2012
KRR1-TAP	W303 *MAT***a** *KRR1-TAP*::natNT2	Koch et al., 2012
ENP1-TAP	MGD353-13D *MAT***a** *ade2, arg4, leu2, trp1, ura3, ENP1*-TAP::*TRP1*	Cellzome, Koch et al., 2012
RIO2-TAP	W303 *MAT***a** *RIO2*-TAP::*HIS3*MX6	Koch et al., 2012
NIP1-TAP	DS1-2b *MAT***a** *his3, leu2, trp1, ura3, NIP1-TAP::TRP1*	Cellzome
RPS3-TAP	W303 *MAT***a** *RPS3*-TAP::natNT2	Koch et al., 2012
YAR1-TAP/*YAR1 RPS3*-Flag/*RPS3*	W303 *MAT***a**/α *YAR1*-TAP::*HIS3*MX4/*YAR1 RPS3*-Flag::natNT2/*RPS3*	this study
YAR1-TAP/*YAR1*-TAP *RPS3*-Flag/*RPS3*	W303 *MAT***a**/α *YAR1*-TAP::*HIS3*MX4/YAR1-TAP::*HIS*MX4 *RPS3*-Flag::natNT2/*RPS3*	this study
YAR1-TAP/*YAR1*-TAP *RPS3*-Flag/*RPS3*-Flag	W303 *MAT***a**/α *YAR1*-TAP::*HIS3*MX4/YAR1-TAP::HISMX4 *RPS3*-Flag::natNT2/*RPS3*-Flag::natNT2	this study
YAR1-TAP/*YAR1*-TAP *RPS3*/*RPS3*	W303 *MAT***a**/α *YAR1*-TAP::*HIS3*MX4/*YAR1*-TAP::*HIS3*MX4	this study
RPS-TAP/*RPS3*-TAP *YAR1*-Flag/*YAR1*	W303 *MAT***a**/α *RPS3*-TAP::*HIS3*MX4/*RPS3*-TAP::natNT2 *YAR1*-Flag::natNT2	this study
PJ69-4A	*trp1-901 leu2-3,112 ura3-52 his3-200 gal4Δ gal80Δ LYS2*::*GAL1-HIS3 GAL2-ADE2 met2*::*GAL7*-lacZ	James et al., 1996

Supplementary Table 3. Yeast plasmids

Name	Relevant Information	Source
pHT4467Δ-*RPS3*	CEN6 (instable), *URA3*, *ADE3*, P*RPS3*, T*ADH1*	this study
pHT4467Δ-*RPS20*	CEN6 (instable), *URA3*, *ADE3*, P*RPS20*, T*ADH1*	this study
YCplac33-*RPS20*	CEN, *URA3,* P*RPS20*, T*ADH1*	this study
pHT4467Δ-*HRR25*	CEN6 (instable), *URA3*, *ADE3*, P*HRR25*, T*ADH1*	this study
YCplac111-*RPS3*	CEN, *LEU2*, P*RPS3*, T*ADH1*	this study
YCplac111-*rps3*-K7A/K10A	CEN, *LEU2*, P*RPS3*, T*ADH1*	this study
YCplac111-*rps3*-K8A/R9A	CEN, *LEU2*, P*RPS3*, T*ADH1*	this study
YCplac111-*rps3*-K7A/K8A/R9A/K10A	CEN, *LEU2*, P*RPS3*, T*ADH1*	this study
YCplac111-*rps3*-K7E	CEN, *LEU2*, P*RPS3*, T*ADH1*	this study
YCplac111-*rps3*-K10D	CEN, *LEU2*, P*RPS3*, T*ADH1*	this study
YCplac111-*rps3*-K7E/K10D	CEN, *LEU2*, P*RPS3*, T*ADH1*	this study
YCplac111-SV40NLS-*RPS3*	CEN, *LEU2*, P*RPS3*, T*ADH1*	this study
YCplac111-SV40NLS-*rps3*-K7A/K10A	CEN, *LEU2*, P*RPS3*, T*ADH1*	this study
YCplac111-SV40NLS-*rps3*-K8A/R9A	CEN, *LEU2*, P*RPS3*, T*ADH1*	this study
YCplac111-SV40NLS-*rps3*-K7A/K8A/R9A/K10A	CEN, *LEU2*, P*RPS3*, T*ADH1*	this study
YCplac22-*RPS3*	CEN, *TRP1*, P*RPS3*, T*ADH1*	this study
YCplac22-*rps3*-K7A/K10A	CEN, *TRP1*, P*RPS3*, T*ADH1*	this study
YCplac22-*rps3*-K8A/R9A	CEN, *TRP1*, P*RPS3*, T*ADH1*	this study
YCplac22-*rps3*-K7A/K8A/R9A/K10A	CEN, *TRP1*, P*RPS3*, T*ADH1*	this study
YCplac22-*rps3*-K7E/K10D	CEN, *TRP1*, P*RPS3*, T*ADH1*	this study
YCplac111-*RPS20*	CEN, *LEU2*, P*RPS20*, T*ADH1*	this study
YCplac111-*rps20*-D113K/E115K	CEN, *LEU2*, P*RPS20*, T*ADH1*	this study
YCplac22-*RPS20*	CEN, *TRP1*, P*RPS20*, T*ADH1*	this study
YCplac22-*rps20*-D113K/E115K	CEN, *TRP1*, P*RPS20*, T*ADH1*	this study
YCplac111-*LTV1*	CEN, *LEU2*, P*LTV1*, T*ADH1*	this study
YCplac111-*ltv1*-S336A	CEN, *LEU2*, P*LTV1*, T*ADH1*	this study
YCplac111-*ltv1*-S339A	CEN, *LEU2*, P*LTV1*, T*ADH1*	this study
YCplac111-*ltv1*-S342A	CEN, *LEU2*, P*LTV1*, T*ADH1*	this study
YCplac111-*ltv1*-S336A/S339A	CEN, *LEU2*, P*LTV1*, T*ADH1*	this study
YCplac111-*ltv1*-S339A/S342A	CEN, *LEU2*, P*LTV1*, T*ADH1*	this study
YCplac111-*ltv1*-S336A/S339A/S342A	CEN, *LEU2*, P*LTV1*, T*ADH1*	this study
YCplac111-*ltv1*-S344A/S345A/S346A	CEN, *LEU2*, P*LTV1*, T*ADH1*	this study
YCplac111-*ltv1*-S342A/S344A/S345A/S346A	CEN, *LEU2*, P*LTV1*, T*ADH1*	this study
YCplac111-*ltv1*-S336A/S339A/ S342A/S344A/S345A/S346A	CEN, *LEU2*, P*LTV1*, T*ADH1*	this study
YCplac111-*ltv1*-S336E/S339E/S342E	CEN, *LEU2*, P*LTV1*, T*ADH1*	this study
YCplac111-*ltv1*-S336A/S339A/S342A/S344E/S345E/S346E	CEN, *LEU2*, P*LTV1*, T*ADH1*	this study
YCplac111-*ltv1*-S336E/S339E/S342E/ S344E/S345E/S346E	CEN, *LEU2*, P*LTV1*, T*ADH1*	this study
YCplac111-*LTV1*-yEGFP	CEN, *LEU2*, P*LTV1*, T*ADH1*, C-terminal $(GA)_5$-yEGFP	this study
YCplac111-*ltv1*-S339A/S342A-yEGFP	CEN, *LEU2*, P*LTV1*, T*ADH1,* C-terminal $(GA)_5$-yEGFP	this study
YCplac111-*ltv1*-S336A/S339A/S342A-yEGFP	CEN, *LEU2*, P*LTV1*, T*ADH1,* C-terminal $(GA)_5$-yEGFP	this study
YCplac111-*ltv1*-S336A/S339A/S342A/S345A/S346A-yEGFP	CEN, *LEU2*, P*LTV1*, T*ADH1,* C-terminal $(GA)_5$-yEGFP	this study
YCplac111-*ltv1*ΔNES-yEGFP	CEN, *LEU2*, P*LTV1*, T*ADH1,* C-	this study

	terminal $(GA)_5$-yEGFP	
pGAL111-*LTV1*	CEN, *LEU2*, P*GAL1*, T*ADH1*	this study
pGAL111-*ltv1*-S336A	CEN, *LEU2*, P*GAL1*, T*ADH1*	this study
pGAL111-*ltv1*-S339A	CEN, *LEU2*, P*GAL1*, T*ADH1*	this study
pGAL111-*ltv1*-S342A	CEN, *LEU2*, P*GAL1*, T*ADH1*	this study
pGAL111-*ltv1*-S336A/S339A	CEN, *LEU2*, P*GAL1*, T*ADH1*	this study
pGAL111-*ltv1*-S339A/S342A	CEN, *LEU2*, P*GAL1*, T*ADH1*	this study
pGAL111-*ltv1*-S336A/S339A/S342A	CEN, *LEU2*, P*GAL1*, T*ADH1*	this study
pGAL111-*ltv1*-S344A/S345A/S346A	CEN, *LEU2*, P*GAL1*, T*ADH1*	this study
pGAL111-*ltv1*- S336A/S339A/S342A/ S344A/S345A/S346A	CEN, *LEU2*, P*GAL1*, T*ADH1*	this study
pGAL111-*ltv1*-S336E/S339E/S342E	CEN, *LEU2*, P*GAL1*, T*ADH1*	this study
pGAL111-*ltv1*- S336A/S339A/S342A/S344E/S345E/S346E	CEN, *LEU2*, P*GAL1*, T*ADH1*	this study
pGAL111-*ltv1*- S336E/S339E/S342E/ S344E/S345E/S346E	CEN, *LEU2*, P*GAL1*, T*ADH1*	this study
pGAG4ADC111-*YAR1*	CEN, *LEU2*, P*ADH1*, T*ADH1*, C-terminal $(GA)_5$-G4AD	this study
pGAG4ADC111-*LTV1*	CEN, *LEU2*, P*ADH1*, T*ADH1*, C-terminal $(GA)_5$-G4AD	this study
pGAG4ADC111-*LTV1*(57-463)	CEN, *LEU2*, P*ADH1*, T*ADH1*, C-terminal $(GA)_5$-G4AD	this study
pGAG4ADC111-*LTV1*(120-463)	CEN, *LEU2*, P*ADH1*, T*ADH1*, C-terminal $(GA)_5$-G4AD	this study
pGAG4ADC111-*LTV1*(218-463)	CEN, *LEU2*, P*ADH1*, T*ADH1*, C-terminal $(GA)_5$-G4AD	this study
pGAG4ADC111-*LTV1*(1-219)	CEN, *LEU2*, P*ADH1*, T*ADH1*, C-terminal $(GA)_5$-G4AD	this study
pGAG4ADC111-*LTV1*(1-122)	CEN, *LEU2*, P*ADH1*, T*ADH1*, C-terminal $(GA)_5$-G4AD	this study
pGAG4ADC111-*LTV1*(1-105)	CEN, *LEU2*, P*ADH1*, T*ADH1*, C-terminal $(GA)_5$-G4AD	this study
pGAG4ADC111-*LTV1*(57-219)	CEN, *TRP1*, P*ADH1*, T*ADH1*, C-terminal $(GA)_5$-G4AD	this study
pGAG4ADC111-*LTV1*(57-122)	CEN, *LEU2*, P*ADH1*, T*ADH1*, C-terminal $(GA)_5$-G4AD	this study
pGAG4ADC111-*LTV1*(57-105)	CEN, *LEU2*, P*ADH1*, T*ADH1*, C-terminal $(GA)_5$-G4AD	this study
pGAG4ADC111-*LTV1*(105-219)	CEN, *LEU2*, P*ADH1*, T*ADH1*, C-terminal $(GA)_5$-G4AD	this study
pGAG4ADC111-*ENP1*	CEN, *LEU2*, P*ADH1*, T*ADH1*, C-terminal $(GA)_5$-G4AD	this study
pGAG4BDC22-*RPS3*	CEN, *TRP1*, P*ADH1*, T*ADH1*, C-terminal $(GA)_5$-G4BD	this study
pGAG4BDC22-RPS3(1-198)	CEN, *TRP1*, P*ADH1*, T*ADH1*, C-terminal $(GA)_5$-G4BD	this study
pGAG4BDC22-*RPS3*(1-95)	CEN, *TRP1*, P*ADH1*, T*ADH1*, C-terminal $(GA)_5$-G4BD	this study
pGAG4BDC22-*RPS3*(1-90)	CEN, *TRP1*, P*ADH1*, T*ADH1*, C-terminal G4DBD	this study
pGAG4BDC22-*RPS3*(1-30)	CEN, *TRP1*, P*ADH1*, T*ADH1*, C-terminal $(GA)_5$-G4BD	this study
pGAG4BDC22-*RPS3*(1-15)	CEN, *TRP1*, P*ADH1*, T*ADH1*, C-terminal G4DBD	this study
pGAG4BDC22-*RPS3*(11-95)	CEN, *TRP1*, P*ADH1*, T*ADH1*, C-terminal $(GA)_5$-G4BD	this study
pGAG4BDC22-*RPS3*(15-95)	CEN, *TRP1*, P*ADH1*, T*ADH1*, C-terminal $(GA)_5$-G4BD	this study

pGAG4BDC22-*RPS3*(15-240)	CEN, *TRP1*, P*ADH1*, T*ADH1,* C-terminal (GA)$_5$-G4BD	this study
pGAG4BDC22-*RPS3*(30-240)	CEN, *TRP1*, P*ADH1*, T*ADH1,* C-terminal (GA)$_5$-G4BD	this study
pGAG4BDC22-*RPS3*(95-240)	CEN, *TRP1*, P*ADH1*, T*ADH1,* C-terminal (GA)$_5$-G4BD	this study

P and T denote promoter and terminator, respectively.

Supplementary Table 4. *E. coli* expression plasmids

**Name**	**Relevant Information**	**Source**
pETDuet-1-His6-Yar1	amp^r, T7 promoter/*lac* operator	Koch et al., 2012
pETDuet-1-Flag-Yar1	amp^r, T7 promoter/*lac* operator	this study
pETDuet-1-Yar1/Rps3-His6	amp^r, T7 promoter/*lac* operator	this study
pETDuet-1-Yar1/Rps3(1-95)-His6	amp^r, T7 promoter/*lac* operator	this study
pETDuet-1-Yar1(8-153)/Rps3(1-95)-His6	amp^r, T7 promoter/*lac* operator	this study
pETDuet-1-Yar1(15-200)/Rps3-His6	amp^r, T7 promoter/*lac* operator	this study
pETDuet-1-Yar1(24-200)/Rps3-His6	amp^r, T7 promoter/*lac* operator	this study
pETDuet-1-Yar1(1-150)/Rps3-His6	amp^r, T7 promoter/*lac* operator	this study
pETDuet-1-Yar1(1-137)/Rps3-His6	amp^r, T7 promoter/*lac* operator	this study
pETDuet-1-Yar1(1-119)/Rps3-His6	amp^r, T7 promoter/*lac* operator	this study
pETDuet-1-His6-Rps3/Yar1	amp^r, T7 promoter/*lac* operator	this study
pETDuet-1-His6-Rps3(1-95)/Yar1	amp^r, T7 promoter/*lac* operator	this study
pETDuet-1-His6-Rps3(16-240)/Yar1	amp^r, T7 promoter/*lac* operator	this study
pETDuet-1-His6-Rps3/Ltv1-Flag	amp^r, T7 promoter/*lac* operator	this study
pETDuet-1-His6-Rps20/Ltv1-Flag	amp^r, T7 promoter/*lac* operator	this study
pETDuet-1-His6-Rps20/Ltv1(1-122)-Flag	amp^r, T7 promoter/*lac* operator	this study
pETDuet-1-His6-Rps20/Ltv1(1-217)-Flag	amp^r, T7 promoter/*lac* operator	this study
pETDuet-1-His6-Rps20/Ltv1(218-463)-Flag	amp^r, T7 promoter/*lac* operator	this study
pETDuet-1-His6-Rps20/Ltv1(310-463)-Flag	amp^r, T7 promoter/*lac* operator	this study
pET15b-Ltv1-Flag	amp^r, T7 promoter/*lac* operator	this study
pET15b-Ltv1(S336A/S339A/S342A>A)-Flag	amp^r, T7 promoter/*lac* operator	this study
pET15b-Ltv1(S6>A)-Flag	amp^r, T7 promoter/*lac* operator	this study
pET15b-Enp1-Flag-TEV-ProteinA	amp^r, T7 promoter/*lac* operator	this study
pProEx-GST-TEV-Hrr25	amp^r, TRC promoter/*lac* operator	this study

Supplementary References

1. Koch, B. *et al.* Yar1 protects the ribosomal protein Rps3 from aggregation. *J. Biol. Chem.* **287,** 21806–21815 (2012).
2. Strunk, B. S. *et al.* Ribosome assembly factors prevent premature translation initiation by 40S assembly intermediates. *Science* **333,** 1449–1453 (2011).
3. Granneman, S., Petfalski, E., Swiatkowska, A. & Tollervey, D. Cracking pre-40S ribosomal subunit structure by systematic analyses of RNA-protein cross-linking. *EMBO J.* **29,** 2026–2036 (2010).
4. Pettersen, E. F. *et al.* UCSF Chimera--a visualization system for exploratory research and analysis. *J. Comput. Chem.* **25,** 1605–1612 (2004).
